# A unique Malpighian tubule architecture in *Tribolium castaneum* informs the evolutionary origins of systemic osmoregulation in beetles

**DOI:** 10.1073/pnas.2023314118

**Published:** 2021-03-30

**Authors:** Takashi Koyama, Muhammad Tayyib Naseem, Dennis Kolosov, Camilla Trang Vo, Duncan Mahon, Amanda Sofie Seger Jakobsen, Rasmus Lycke Jensen, Barry Denholm, Michael O’Donnell, Kenneth Veland Halberg

**Affiliations:** ^a^Department of Biology, Section for Cell and Neurobiology, University of Copenhagen, DK-2100 Copenhagen, Denmark;; ^b^Department of Biology, McMaster University, Hamilton, ON L8S 4K1, Canada;; ^c^Department of Biological Sciences, California State University San Marcos, San Marcos, CA 92069;; ^d^Centre for Discovery Brain Sciences, University of Edinburgh, Edinburgh EH8 9AG, United Kingdom

**Keywords:** *Tribolium castaneum*, Malpighian tubule, diuretic hormone, osmoregulation, secondary cell

## Abstract

Maintaining internal salt and water balance in response to fluctuating external conditions is essential for animal survival. This is particularly true for insects as their high surface-to-volume ratio makes them highly susceptible to osmotic stress. However, the cellular and hormonal mechanisms that mediate the systemic control of osmotic homeostasis in beetles (Coleoptera), the largest group of insects, remain largely unidentified. Here, we demonstrate that eight neurons in the brain of the red flour beetle *Tribolium castaneum* respond to internal changes in osmolality by releasing diuretic hormone (DH) 37 and DH47—homologs of vertebrate corticotropin-releasing factor (CRF) hormones—to control systemic water balance. Knockdown of the gene encoding the two hormones (*Urinate*, *Urn8*) reduces Malpighian tubule secretion and restricts organismal fluid loss, whereas injection of DH37 or DH47 reverses these phenotypes. We further identify a CRF-like receptor, Urinate receptor (Urn8R), which is exclusively expressed in a functionally unique secondary cell in the beetle tubules, as underlying this response. Activation of Urn8R increases K^+^ secretion, creating a lumen-positive transepithelial potential that drives fluid secretion. Together, these data show that beetle Malpighian tubules operate by a fundamentally different mechanism than those of other insects. Finally, we adopt a fluorescent labeling strategy to identify the evolutionary origin of this unusual tubule architecture, revealing that it evolved in the last common ancestor of the higher beetle families. Our work thus uncovers an important homeostatic program that is key to maintaining osmotic control in beetles, which evolved parallel to the radiation of the “advanced” beetle lineages.

## Significance

Beetles are the most diverse animal group on the planet. Their evolutionary success suggests unique physiological adaptations in overcoming water stress, yet the mechanisms underlying this ability are unknown. Here we use molecular genetic, electrophysiology, and behavioral studies to show that a group of brain neurons responds to osmotic disturbances by releasing diuretic hormones that regulate salt and water balance. These hormones bind to their receptor exclusively localized to a unique secondary cell in the Malpighian tubules to modulate fluid secretion and organismal water loss. This tubule architecture, common to all higher beetle families, is novel within the insects, and provides an important clue to the evolutionary success of the beetles in colonizing an astounding range of habitats on Earth.

Animals must continuously defend against perturbations in internal osmolality as they interact with their external environment. Fluctuations in extracellular fluid (ECF) solute concentrations cause water to flow across cell membranes until a new osmotic equilibrium is reached. These changes result in altered cell volume and ionic strength, which can severely affect the physical integrity and biological activity of cells and tissues. For this reason, systemic osmoregulation is essential to organismal survival and is accordingly under tight control. However, the cellular mechanisms and interorgan communication networks that mediate the systemic control of osmotic homeostasis in different animal Phyla remain largely unexplored.

The evolutionary success of insects is tightly coupled with their ability to regulate ion and water balance as their small size and large surface-to-volume ratio make them highly susceptible to osmotic stress. The main osmoregulatory organs in insects are the Malpighian (renal) tubules (MTs), which, along with the hindgut, constitute the functional analog of the vertebrate kidney (1). In the fruit fly *Drosophila melanogaster*, the integrated actions of the MTs rely on the spatial segregation of cation and anion transport into two physiologically distinct cell types, the principal cell (PC) and the secondary (stellate) cell (SC). Whereas the large PCs mediate electrogenic cation transport, the smaller SCs control the anion conductance and water transport (2–5). Both cell types are under complex and independent neuroendocrine control, with PCs receiving regulatory input from diuretic hormone (DH) 31, Capa, and DH44 (6–8), while SC activity is modulated by kinin and tyramine signaling (9, 10). Remarkably, the hormonal signals diagnostic of PC and SC functions map to similar cell types across most of the holometabolous insects, suggesting that this two-cell–type model and associated neurohormone signaling is both ancient and conserved (11). Yet, a striking exception to this epithelial model was found in the large Order of Coleoptera, the beetles, as members of this group possess a MT architecture separate from that of all other insects. Beetles appear to lack kinin signaling altogether, while both Capa and DH31 activity is confined to a small population of PCs (11). Moreover, genomic and systematic evidence suggests that other signaling systems typically involved in controlling diuresis in other insects are either secondarily lost or greatly expanded (12–14). Together, these results suggest that the MTs of beetles—an insect Order containing almost 40% of insect biodiversity (15, 16)—function in a fundamentally different way than in all other insects (11). Unraveling the homeostatic mechanisms that govern systemic osmoregulation in beetles is important, not only because it offers insights into the evolutionary success of the most species-rich group of animals on the planet, but also because it could help identify novel beetle-specific pest control solutions.

Here, we report that corticotropin releasing factor-like (CRF-like) DH signaling plays a central role in controlling systemic osmoregulation in the red flour beetle, *Tribolium castaneum*. Using molecular genetics, imaging, electrophysiology, organ assays, and behavioral studies, we show that a group of neurons in the brain responds bidirectionally to changes in ECF osmolality by releasing DH37 and DH47 hormones into circulation to remotely control tubule secretion and systemic water balance. We identify a CRF-like receptor, named Urinate receptor (Urn8R), which uniquely localizes to a type of SC interspersed along the MTs as underlying this response. Activation of Urn8R increases the luminal directed K^+^ flux most likely through SCs, which creates a lumen-positive transepithelial potential (TEP) that drives fluid secretion via a cyclic AMP (cAMP)-dependent mechanism. Finally, to test the evolutionary origins of this MT architecture, we mapped the subcellular location of DH37 and DH47 action in MTs from strategically chosen representatives of all major beetle families (covering >70% of beetle biodiversity) to provide an unprecedented phylogenetic overview of beetle tubule function and control. Altogether, our work uncovers an important homeostatic program that is key to maintaining body fluid balance in beetles, a program operating via a two-cell–type model unique to this group of insects that evolved alongside the rapid diversification of the higher beetle families.

## Results

### Orphan G Protein-Coupled Receptors Show Enriched Expression in Tubules.

**Fig. 1.**
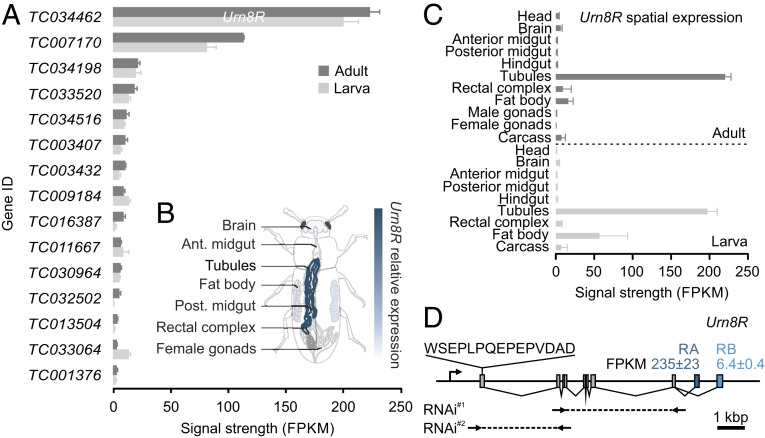
Transcriptomic mapping of GPCR gene expression in MTs of *T. castaneum*. (*A*) RNA-seq analyses listing transcript abundance (FPKM) of all genes predicted to encode a GPCR with significantly higher expression in larval and adult MTs relative to the whole larva and adult animal, respectively. From this list, *TC034462* shows the highest expression, which we propose to name *Urn8R*. (*B*) Anatomical map of adult *T. castaneum* with superimposed heat-map of *Urn8R* expression across different tissues. (*C*) The spatial expression pattern of *Urn8R* indicates that it almost exclusively expressed in tubules of both larvae and adults. (*D*) Exon map of the *Urn8R* gene. The receptor is predicted to be expressed in two isoforms with -*RA* (235 ± 23 FPKM) showing much higher expression than -*RB* (6.4 ± 0.4 FPKM). The amino acid sequence used to raise an anti-Urn8R–specific antibody, as well as the sequences targeted for RNAi knockdown (RNAi^#1^: base pairs 279 to 896; RNAi^#2^: base pairs 52 to 273) are indicated. RNAi^#1^ produced the most effective knockdown (87% ± 0.04 SEM; *n* = 5) and was therefore used for all subsequent experiments.

To gain unbiased insights into the hormonal systems that control MT function in beetles, we adopted an RNA-sequencing (RNA-seq) approach in which we generated an authoritative overview of gene expression across all major tissues from both larval and adult *T. castaneum*. Using these data, we performed hand-searches on all genes predicted as G protein-coupled receptors (GPCRs) in the *T. castaneum* genome (14), and looked for transcript enrichment in the MTs relative to that of the whole animal. Through this transcriptomic approach, we identified 15 GPCR genes that showed significantly higher expression (fragments per kilobase of exon model per million reads mapped; FPKM) in MTs than in the whole animal, revealing that the *T. castaneum* tubule is an important signaling hub capable of responding to a variety of different signals. Of these 15 GPCR genes, *TC034462* (a gene we propose to name *Urn8R*) shows the highest expression (Fig. 1*A*); transcript abundance of *Urn8R* in MTs was verified by qRT-PCR (*SI Appendix*, Fig. S1*A*). Looking at the transcriptional profile of this orphan receptor, we further discovered that it was almost exclusively expressed in the larval and adult MTs (Fig. 1 *B* and *C*) and of the two splice variants predicted (-*RA* and -*RB*), *Urn8RA* was the dominant isoform expressed (Fig. 1*D*). Based on tissue enrichment criteria and the spatial expression pattern of *Urn8R*, we focused our attention on the functional roles of this gene in tubule physiology.

### Deorphanization of a CRF-like Receptor.

To discern the structural properties of Urn8R, we performed homology modeling and three-dimensional structure predictions of the deduced amino acid sequence using the publicly available GPCRM Structure Modeling Server (17) to identify the putative transmembrane domains and overall topology of the receptor. This analysis confirmed that Urn8R is a seven-transmembrane receptor (*SI Appendix*, Fig. S1*B*) that shows homology to the CRF-like receptor family (49% amino acid sequence identity with *D. melanogaster* DH44 receptor 1; E-value 4e-119) and therefore belongs to a class B secretin-like subfamily of GPCRs. These findings are consistent with previous in silico predictions identifying the gene encoding this protein as a candidate CRF-like receptor (14).

Next, we sought to identify the endogenous ligands of the receptor by using a reverse pharmacological approach. To this end, we independently cloned and heterologously expressed the two *Urn8R* isoforms (*Urn8RA* and -*RB*) into competent Chinese hamster ovary (CHO) cells (*SI Appendix*, Fig. S1*C*), which allowed quantification of bioluminescence responses following activation of the heterologous receptor (18). Testing a small peptide library representing eight different neuropeptide families, we found that both receptor isoforms were strongly activated by micromolar concentrations of *D. melanogaster* DH44, as well as the putative *T. castaneum* CRF-like ligands DH37 and DH47 (12) (*SI Appendix*, Fig. S1*D*). None of the other peptides induced significant receptor activation at concentrations up to 10^−6^ M (*SI Appendix*, Fig. S1*D*). Of the two splice variants, Urn8RA showed highest activation by DH37 (EC_50_, 1.6 × 10^−7^ M), and only ∼50% of the maximum response by DH47 (EC_50_, 3.8 × 10^−7^ M), indicating that DH47 is a partial agonist of this isoform. Conversely, Urn8RB showed higher activation by DH47 (EC_50_, 3.4 × 10^−7^ M) and a mere ∼30% of the maximum activity by DH37 (EC_50_, 4.5 × 10^−7^ M) (*SI Appendix*, Fig. S1 *E*–*H*).

Having identified the endogenous ligands of the receptor, we sought to identify the intracellular signaling pathways activated by Urn8R stimulation. In other insects, there is a broad consensus that CRF ligand-receptor binding induces adenylate cyclase protein kinase A activation and a rapid production of the second messenger cAMP (6, 19, 20). We therefore examined agonist-stimulated signaling of Urn8R in MTs via intracellular cAMP accumulation using an ultrasensitive FRET-based LANCE ULTRA method. This technique is an immunoassay based on the competition between a Europium-labeled cAMP tracer and sample cAMP for binding sites on cAMP-specific antibody labeled with a fluorescent dye. These experiments showed that in dissected tubules treated with either DH37 or DH47 induce a strong receptor activation in the nanomolar range, with DH37 producing a larger tissue response (EC_50_ values of 3.65 × 10^−9^ M) compared to DH47 (EC_50_, 6.13 × 10^−9^ M) as measured by cAMP production per tubule (*SI Appendix*, Fig. S1 *I* and *J*). Taken together, our data suggest that Urn8R is a *T. castaneum* CRF-like receptor that is activated by its endogenous ligands DH37 and DH47 that signals through cAMP.

### Urn8R Plays a Critical Role in Regulating MT Function.

**Fig. 2.**
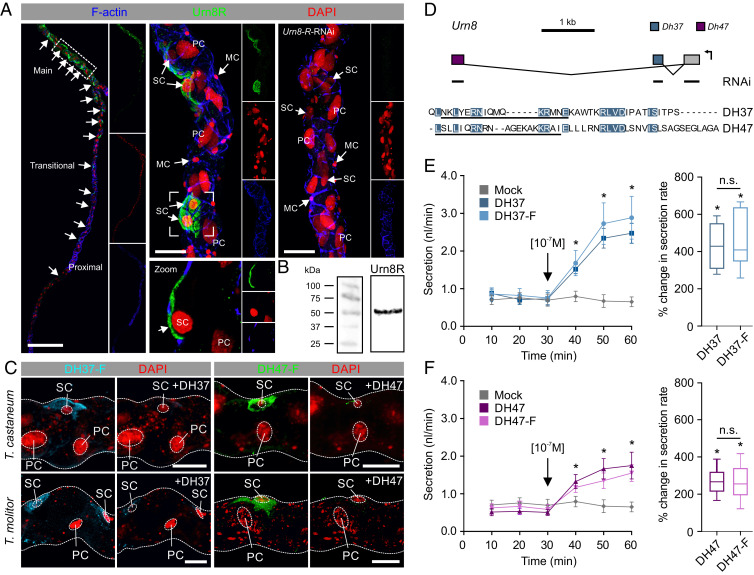
The Urn8 receptor localizes to a secondary cell type in MTs and is activated by its endogenous ligands DH37 and DH47. (*A*) Urn8R is expressed in a subpopulation of cells throughout the “free” part of the *T. castaneum* MT (small arrows); there are 53 ± 2 (*n* = 8) cells per tubule. Subcellular localization of Urn8R reveals exclusive expression to the basolateral membrane of a small-nucleated SC type (small arrows). MTs from animals injected with dsRNA targeting the Urn8 receptor (*Urn8R*-*RNAi*) showed a complete loss of immunoreactivity confirming specificity of the antibody. (Scale bars, *Left*, 150 μm; *Middle* and *Right*, 30 μm.) (*B*) Western blot analysis of protein extracts from *T. castaneum* tubules showing that the anti-Urn8R antiserum recognizes a protein of a size of approximately 50 to 55 kDa, consistent with the predicted size of the receptor (51.6 kDa for the dominant -RA isoform, arrow). (*C*) Application of fluorophore-coupled receptor agonists DH37-F and DH47-F (10^−7^ M) to *T. castaneum* and *T. molitor* MTs. Specific and displaceable binding to the SCs is observed, as competitive inhibition with “cold” unlabeled ligands DH37 and DH47 (10^−5^ M) almost fully abolished the fluorescent signal. (Scale bars, 20 μm.) (*D*) Exon map of the *T. castaneum Urn8* (*TC030022*) gene encoding the two predicted Urn8 ligands, DH37 and DH47, produced by alternative splicing of two separate exons to a common 5′ exon encoding the signal peptide (12). The regions targeted for RNAi knockdown (either common or ligand-specific sequences) and the amino acid sequences used to raise ligand-specific antibodies are indicated. Amino acids shared between the two ligands are highlighted in blue. (*E* and *F*) Both the labeled and unlabeled ligands significantly stimulate fluid secretion rates compared to unstimulated (artificial hemolymph only; mock) MTs of *T. molitor* (paired-sample *t* test, *n* = 10, *P < 0.05). Black arrows indicate time of peptide application. DH37 induces a significantly higher percent change in fluid secretion compared to DH47 (unpaired-sample *t* test, *n* = 10, *P < 0.05). Fluorophore coupling does not significantly affect the functional efficacy of the peptides (unpaired-sample *t* test, *n* = 10, n.s. *P* > 0.05). Values are expressed as mean ± SEM.

To dissect the molecular mechanism underpinning Urn8-mediated control of tubule function, we immunolocalized Urn8R to the tubule epithelium of *T. castaneum*. Surprisingly, these data showed that the receptor exclusively localizes to the basolateral membrane of a small-nucleated, yet morphologically indistinct, SC-type throughout the main segment of the “free” tubule (53 ± 2 SC per tubule, *n* = 8), suggesting that SCs have adopted Urn8 signaling to the exclusion of other cell types (Fig. 2*A*). The SC identity of this *Urn8R*-expressing cell type was verified by colocalization of the Tiptop transcription factor, which is known to control SC differentiation in other insects (21) (*SI Appendix*, Fig. S2*A*). The fact that the Urn8 pathway is confined exclusively to SCs in *T. castaneum* is in contrast to that of all other insects studied to date in which this hormonal circuit is diagnostic of PC activity, the majority cell type (22–24). Consistent with the RNA-seq data, we also observed specific Urn8R immunopositive neurons in the adult brain, while tissues such as the fat body and carcass showed no detectable immunoreactivity (*SI Appendix*, Fig. S2*B*). Specificity of the antibody was verified by the lack of staining in MTs from *Urn8R*-silenced animals, and by the fact that the molecular weight of the protein corresponds to the predicted size of the receptor (Fig. 2 *A* and *B*). Next, we examined if the putative receptor ligands DH37 and DH47 also bind to Urn8R in live tissue. To do this, we generated fluorophore-coupled DH37/DH47 analogs (DH37-F/DH47-F) and applied them in combination with a novel ligand–receptor binding assay (11). This approach allows direct visualization of ligand–receptor interactions and revealed that both DH37-F and DH47-F bind to basolateral membranes of SCs in MTs from *T. castaneum*, as well as in tubules from a closely related species *Tenebrio molitor*; specificity of binding was confirmed by the displacement of signal by coapplication of “cold” unlabeled peptides (Fig. 2*C*). These data confirm that DH37 and DH47 bind to Urn8R on the SC basal membrane in *T. castaneum*, but also suggest that the MT organization and the mode of action of Urn8 signaling are conserved among tenebrionid beetles.

To determine how Urn8R activation in SCs affects tubule physiology, we quantified changes in tubule output using the Ramsay fluid secretion assay (11). Given that the small size of *T. castaneum* tubules make them unsuitable for this assay, we rationalized that the larger *T. molitor* MTs would be more amenable to physiological studies (25). Importantly, orthologs of both DH ligands have been isolated by mass spectrometry from *T. molitor* (26, 27), revealing that they share 73% or 100% amino acid sequence identities with DH37 and DH47 from *T. castaneum* (Fig. 2*D*), respectively (12). Applying DH37 or DH47 to *T. molitor* tubules ex vivo—at a concentration shown to cause maximum receptor occupation in *T. castaneum* (*SI Appendix*, Fig. S1)—we found that both ligands significantly stimulate fluid secretion when applied individually compared to artificial hemolymph control (Fig. 2 *E* and *F*). Moreover, we observed that DH37 induces a significantly higher response compared to DH47, and the fluorophore-coupled analogs were equipotent to the unlabeled ligands in both the secretion and cell-based assays (Fig. 2 *E* and *F* and *SI Appendix*, Fig. S1 *E*–*H*). In light of the ability of DH37 and DH47 to stimulate MT secretion, we propose to name the gene encoding these two ligands *urinate* (*Urn8*) (Fig. 2*D*). In sum, our results indicate that the *T. castaneum* (and *T. molitor*) tubule is a functionally heterogenic tissue in which Urn8R activation, exclusively in SCs, functions to stimulate the tissue to increase urine production.

### Urn8 Signaling Modulates Regional and Cell-Specific Ion Flux.

**Fig. 3.**
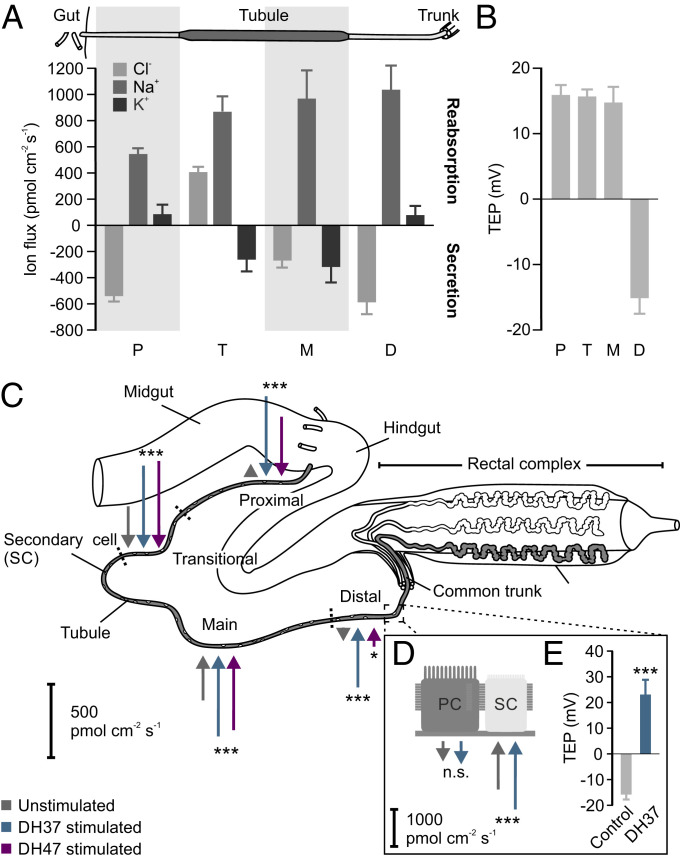
Urn8 signaling modulates regional and cell-specific ion transport rates in tubules. (*A*) Quantitative SIET data showing Cl^−^, Na^+^, and K^+^ fluxes in the proximal (P), transitional (T), main (M), and distal (D) regions of the “free” tubules from *T. molitor*. The relative length of the different functional regions of the tubule is not drawn to scale. Positive values indicate ion reabsorption (from tubule lumen to hemolymph) and negative values indicate secretion (from hemolymph to tubule lumen). (*B*) TEP voltage differences in the functionally distinct regions. (*C*) Schematic diagram summarizing regional effects of DH37 and DH47 stimulation on K^+^ secretion in all four regions of the tubule as measured by SIET. Arrows directed toward the tissue denote net secretion, while arrows directed outward indicate reabsorption. The length of the arrows corresponds to the average ion flux in each region. Both DH37 and DH47 increase net K^+^ secretion in all four regions.(one-way ANOVA, *n* = 6, *P < 0.05, ***P < 0.001). (*D*) Cell-specific measurements indicate that DH37-induced changes in K^+^ secretion are likely mediated by the SCs (Student’s *t* test, *n *= 3, n.s. *P* > 0.05, ***P < 0.001) which (*E*) reverses the lumen-negative potential in the distal segment (Student’s *t *test, *n *= 3, ***P < 0.001). All data are presented as mean ± SEM.

The hormonally induced change in tubule secretion points to a stimulatory role of Urn8R in transepithelial ion movement. Yet, whether the ion transport competencies of the tubule epithelium are uniformly distributed or alternatively spatially segregated into different functional domains remains unresolved. To distinguish between these two models, we used a powerful noninvasive method called the scanning ion-selective electrode technique (SIET) to map potential region and cell-specific ion flux rates. Surprisingly, applying this method to *T. molitor* tubules, we detected clear region-specific differences in cation and anion handling across the tubule (Fig. 3*A*). The morphologically defined proximal and distal regions (thinner and more translucent) were found to reabsorb K^+^ and secrete Cl^−^; an electrophysiological signature associated with fluid reabsorption and base recovery in other insects (28, 29). In contrast, the large main segment (thicker and more pigmented) was discovered to secrete both K^+^ and Cl^−^, suggesting that this domain is likely the major fluid-producing region of the tubule. Although morphologically indistinct, the main segment could be further subdivided near the proximal–main segment boundary, as this transitional region was uniquely defined by secreting K^+^ but reabsorbing Cl^−^. In all regions of the tubule, we detected a prominent net reabsorption of Na^+^, indicating that this ion is unlikely to be involved in driving fluid secretion directly (Fig. 3*A*).

Next, we wondered how these region-specific differences in ion flux affected the combined voltage across the epithelium. Performing TEP measurement in the different domains, we discovered that the TEP was consistently ∼15 mV lumen-positive compared to the bathing saline in the proximal, transitional and main segments. Yet, the TEP reversed in the distal region becoming as much as 15 mV lumen-negative relative to bath (Fig. 3*B*). This supports a model in which this segment acts in concert with the rectal complex to return water reabsorbed from the rectal lumen back into the hemolymph (30, 31). Together, these data emphasize that the tenebrionid tubule is a functionally heterogeneous tissue that contains not only two different secretory cell types, but also at least four physiologically distinct regions.

We then explored how Urn8 signaling modulates the electrophysiological signatures of the epithelium to stimulate tubule secretion. As both our data and previous reports indicate that K^+^ is the principal ion driving tubule secretion in tenebrionid beetles (32), we focused our attention on DH37/DH47-induced changes in K^+^ flux. When tubules were stimulated with DH37 or DH47 independently, the average K^+^ flux almost doubled in the main and transitional segments, suggesting that these are the main fluid-secreting regions of the tubule (Fig. 3*C* and *SI Appendix*, Fig. S3*A*). Yet, at the same time we surprisingly detected a complete reversal of the K^+^ flux in both the distal and proximal segments, implying that these regions are “recruited” during humoral stimulation to increase fluid production. Moreover, we observed a conspicuously larger response upon DH37 stimulation relative to DH47 application in these segments, indicating that the Urn8RA splice variant may be dominantly expressed here due to the isoform-specific ligand–receptor kinetics (*SI Appendix*, Fig. S1).

Conceivably, both paracellular transport and a transcellular route through a small subset of cells could explain the observed changes in tubule secretion. Given that Urn8R localizes exclusively to SCs, however, we rationalized that the task of potassium transport would also be spatially restricted within the tubule. Indeed, when performing our analysis of the tubule at a higher resolution, it became evident that the regional changes in K^+^ conductance was confined to a relatively small number of hot spots throughout the “free” part of the tubule (Fig. 3*D* and *SI Appendix*, Fig. S3 *B* and *C*), consistent with SCs mediating the transepithelial potassium flux. Furthermore, when the tubule was stimulated with DH37, the average K^+^ secretion increased significantly at these hot spots, which, at least in the distal region, associated with the reversal of the lumen-negative TEP (Fig. 3 *D* and *E* and *SI Appendix*, Fig. S3 *B* and *C*). Taken together, our data point to a model in which Urn8 signaling increase primary urine secretion by offering a privileged route for transepithelial K^+^ transport, most likely through SCs.

### Eight Neurons Localized to the *Pars Intercerebralis* Mediate the Effects.

**Fig. 4.**
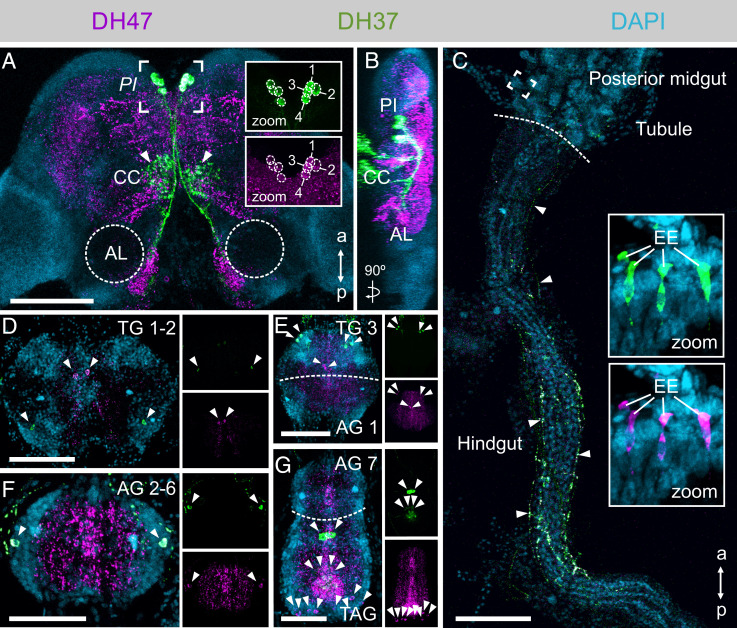
Anatomy of the DH37- and DH47-producing neurons in adult *T. castaneum.* (*A* and *B*). DH37 and DH47 are coexpressed in four pairs of neurons in the *PI* region of the brain. (*Insets* 1.5× magnification.) The *PI* neurons arborize to the corpora cardiaca (CC, arrowheads) and to the more posterior part of the brain near the antennal lobes (AL). (Scale bar, 100 μm.) (*C*) DH37^+^/DH47^+^ neurons further innervate the posterior-most part of the midgut and project along the hindgut to the anterior part of the rectal complex (arrowheads). Both peptides are also expressed in EEs of the posterior midgut (*Insets*, 10× magnification). (Scale bar, 100 μm.) (*D–G*) Several distinct pairs of DH37^+^ and DH47^+^ neurons are found in the thoracic (TG), abdominal (AG), and terminal (TAG) ganglia of the ventral nerve cord (arrowheads). (Scale bars, 50 μm.)

To identify the neuronal circuitry underlying these physiological responses, we raised ligand-specific antibodies against DH37 and DH47 (Fig. 2*D*) and immunolocalized these neuropeptides to both nervous and peripheral tissues (Fig. 4). We found four pairs of DH37 and DH47 immune-positive neurosecretory cells in the ventral midline of the *pars intercerebralis* (*PI*), a region of the insect brain typically populated with neurosecretory cells (33, 34) (Fig. 4 *A* and *B*). These two groups of neurosecretory cells cross the midline of the brain and project their axons posteriorly to leave the brain where they arborize in the contralateral corpus cardiacum, which typically stores and releases hormones into the hemolymph in other insects (35). Some dendritic processes, mainly containing anti-DH47 immunoreactivity, from these neurosecretory cells could moreover be traced to neuropil near the antennal lobes. This observation is consistent with DH47 being identified by direct peptide profiling of this region of the brain (36), and suggests a putative role of urn8 signaling in modulating the olfactory pathway. In the gut, fine DH37^+^ and DH47^+^ neuronal processes were additionally seen to innervate the hindgut region extending from the posterior midgut to the rectal complex, while numerous immune-positive enteroendocrine cells (EE) were detected in the midgut region (Fig. 4*C*). Moreover, several distinct DH37^+^ and DH47^+^ neurons were also found in the central and lateral regions of both the thoracic (TG1-3) and abdominal ganglia (AG1-7) of the ventral nerve cord (Fig. 4 *D*–*G*), suggesting that the Urn8 prohormone can be differentially processed to allow both separate as well as corelease of these peptides to fine-tune Urn8 activity. In sum, our data suggest that DH37 and DH47 peptides are released systemically from neuroendocrine cells in the *PI*, as well as from the ventral nerve cord and midgut EEs to exert their physiological effects.

### Internal Water Abundance Modulates Urn8 Signaling.

**Fig. 5.**
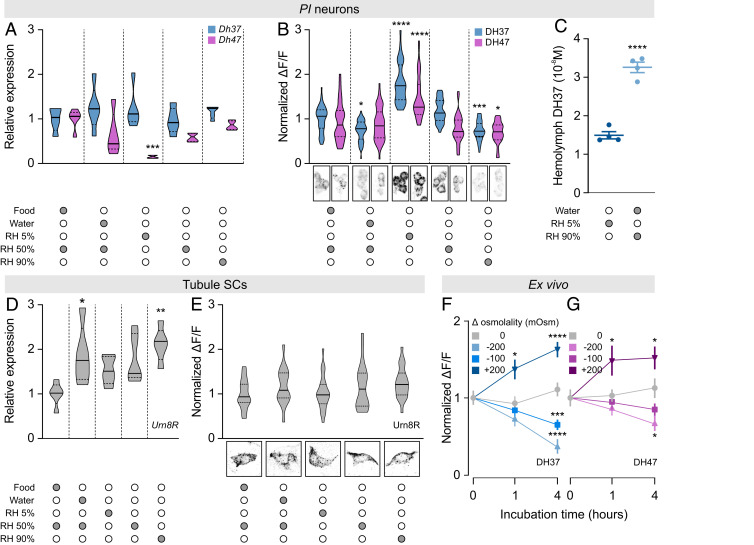
Environmental cues modulating Urn8 signaling activity. (*A* and *B*) Violin plots of brain *Dh37/Dh47* transcript (*n* = 5) and DH37/DH47 peptide (*n* = 40 to 64) levels in *PI* neurons of brains from *T. castaneum* exposed to different environmental conditions. Representative images of DH37 and DH47 immunostaining from each condition are shown below. (*C*) Circulating DH37 levels in hemolymph from *T. molitor* exposed to either low (dark blue) or high (light blue) RH as measured by ELISA (Student's *t* test, *n* = 4, **** *P* < 0.0001). (*D* and *E*) Tubule *Urn8R* transcript (*n* = 5) and Urn8R protein (*n* = 17 to 25) levels from *T. castaneum* exposed to the different environmental conditions. Representative images of Urn8R immunoreactivity are shown below. (*A–B* and *D*–*E*) Significant differences indicate pairwise comparisons between control (food, RH 50%) and a given experimental group (one-way ANOVA, *P < 0.05, **P < 0.01, ***P < 0.001, ****P < 0.0001). (*F* and *G*) DH37 (blue, *F*) and DH47 (magenta, *G*) peptide levels from *T. castaneum* brains (*n* = 14 to 35) cultured ex vivo in different hypo- and hyperosmotic artificial hemolymph (AHL) solutions for 0, 1, and 4 h, respectively (one-way ANOVA, *P < 0.05, ***P < 0.001, ****P < 0001).

The potent activation of tubule secretion by both DH37 and DH47 suggests a role of Urn8 signaling in the homeostatic control of internal ion and water balance. Accordingly, we asked whether the Urn8 circuitry responds to internal cues related to water availability by comparing transcript and protein levels of both ligands and receptor in animals exposed to conditions known to affect hemolymph osmolality (37) (*SI Appendix*, Fig. S4*A*). Apart from a significant reduction in *Dh47* transcript levels during low humidity exposure (RH 5%), the different environmental conditions did not alter *Dh37* or *Dh47* expression in the brain (Fig. 5*A*). However, by measuring hormone retention levels we found that the *PI* neurons are sensitive to changes in internal water availability, given that exposure to desiccating conditions (RH 5%) significantly increased, while high humidity exposure (RH 90%) consistently lowered, the intracellular DH37 and DH47 protein levels relative to control animals. Furthermore, drinking only (water) also induced a small but significant decrease in DH37 immunoreactivity (Fig. 5*B*). These data imply that the *PI* neurons are inactive during periods of water restriction, yet are induced to release DH37 and DH47 neuropeptides at high rates following exposure to conditions that cause fluid overload. To formally test this hypothesis, we quantified circulating DH37 levels in hemolymph from *T. molitor* subjected to either severe desiccation or high humidity conditions using ELISA. We observed a significant increase in circulating DH37 levels in animals subjected to a high humidity environment compared to beetles exposed to desiccating conditions, confirming that the DH peptides are released during periods of excess liquid (Fig. 5*C*). Moreover, consistent with these findings, transcript levels of *Urn8R* in the MTs were significantly increased during high humidity (90% RH) and drinking only (water) conditions, which suggest a potential up-regulation of Urn8 sensitivity in the excretory organs during periods of excess fluid (Fig. 5 *D* and *E*). Intriguingly, changes in immunoreactivity were not detected in neurons of the ventral nerve cord or in the EEs of the posterior midgut (*SI Appendix*, Fig. S4 *B* and *C*), supporting the view that only the *PI* neurons relay information about changes in internal water levels.

To test if the environmentally induced changes in hemolymph osmotic pressure are sensed autonomously by the brain, or alternatively depend on regulatory input from other tissues, we incubated *T. castaneum* brains in artificial hemolymph solutions of different osmolalities and probed each brain for intracellular DH37 and DH47 protein levels. Decreasing osmolality caused robust dose-dependent reductions in both DH37 and DH47 immunoreactivity exclusively in the *PI* neurons, indicating that both neuropeptides are released at high rates during hypotonic conditions. In contrast, increasing extracellular osmolality induced a marked increase in fluorescence, suggesting that both DH37 and DH47 are retained (Fig. 5 *F* and *G*). Together, these data show that DH37 and DH47 release from the *PI* neurons is bidirectionally regulated by external osmolality and that the brain is capable of autonomously reporting both magnitude and polarity of changes in ECF osmolality.

### Silencing *Urn8R* or *Urn8* Expression Improves Desiccation Tolerance.

**Fig. 6.**
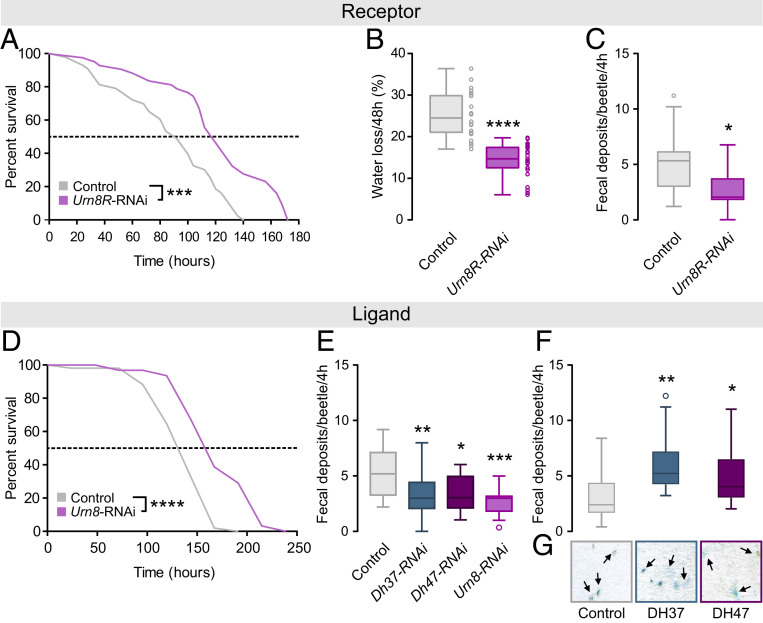
Urn8 signaling regulates systemic water balance. (*A*) Kaplan–Meyer survival function of control (dsRNA targeting *beta*-*lactamase*, *amp*^*R*^) and *Urn8R*-silenced animals. *Urn8R* knockdown animals survive significantly longer than controls during desiccation stress (RH 5%; log-rank test, *n* = 43, ***P < 0.001). (*B*) Gravimetric analyses of control and *Urn8R*-depleted animals. Desiccation-induced water loss is significantly reduced in *Urn8R* knockdown animals compared to control (unpaired Student’s *t* test, *n* = 22 to 25, ****P < 0.0001). (*C*) Defecation rate of *Urn8R* silenced animals is significantly lower than that of controls (unpaired Student’s *t* test, *n* = 18, *P < 0.05). (*D*) Kaplan–Meyer survival function of *Urn8* silenced animals. *Urn8* knockdown animals survive significantly longer than controls during desiccation stress (RH 5%; log-rank test, *n* = 34 to 53, ****P < 0.0001). (*E*) Defecation rate of *Dh37*-/*Dh47*-specific as well as *Urn8* knockdown animals are significantly reduced relative to controls (one-way ANOVA, *n* = 19 to 22, *P < 0.05, **P < 0.01, ***P < 0.001). (*F*) Injection of DH37 or DH47 peptides (final concentration approximately 10^−7^ M) into *Urn8*-depleted animals induce a significant increase in the number of BDP-labeled excreta produced compared to buffer-injected controls (one-way ANOVA, *n* = 37, *P < 0.05, **P < 0.01). (*G*) Representative images (size: 300 × 300 μm) of excreta produced in *E*. DH37 or DH47 injection also results in lighter, less concentrated deposits and in increased fluid excretion (arrows) relative to controls.

To further explore the functional significance of Urn8 signaling in maintaining osmotic homeostasis in vivo, we selectively down-regulated *Urn8R* or *Urn8* expression using RNA interference (RNAi). Given that the rate of water loss is the main factor determining insects’ resistance to dry environments (38, 39), we rationalized that silencing Urn8 signaling in *T. castaneum* might reduce fluid excretion and thus improve desiccation tolerance. Consistent with this hypothesis, *Urn8/Urn8R*-silenced beetles survived significantly longer than control injected animals during severe desiccation, with a median survival of 158 and 116 h, as compared to a median lifespan of 130 and 92 h in control animals, respectively (Fig. 6 *A* and *D*); RNAi efficacy was verified by qRT-PCR and immunocytochemistry showing almost 90% knockdown and complete loss of detectable Urn8 and Urn8R expression (*SI Appendix*, Fig. S5 *A*–*C*). This enhanced survival is likely caused by improved body water retention, since *Urn8R* depleted beetles had a higher wet weight, but a similar dry weight, and a consistently lower rate of water loss relative to control (Fig. 6*B* and *SI Appendix*, Fig. S5*D*). Moreover, using food supplemented with the pH indicator dye Bromophenol blue to obtain colored excreta, we also detected a markedly lower defecation rate in *Urn8R* knockdown beetles (Fig. 6*C*), consistent with the established correlation between tubule activity and intestinal emptying rate in insects (40, 41). Indeed, inactivating the *PI* neurons by silencing *Dh37* and *Dh47* separately or knocking down full-length *Urn8* expression also resulted in the excretion of significantly fewer deposits, whereas injection with DH37 or DH47 hormones induced a marked increase in the number of excreta as compared to mock-injected control (Fig. 6 *E* and *F*). Intriguingly, the deposits made by DH37- or DH47-injected animals were further coupled with a striking increase in fluid excretion, as clearly evidenced by the excess liquid surrounding their excreta, which was never observed in mock-injected animals (Fig. 6*G*). Altogether, our results identify a hormonal circuit consisting of two small groups of osmosensitive neurons, which release DH37/DH47 hormones into circulation to remotely control MT secretion and stabilize internal ion and water balance.

### Evolutionary Scope of a Two-Cell–Type Model.

**Fig. 7.**
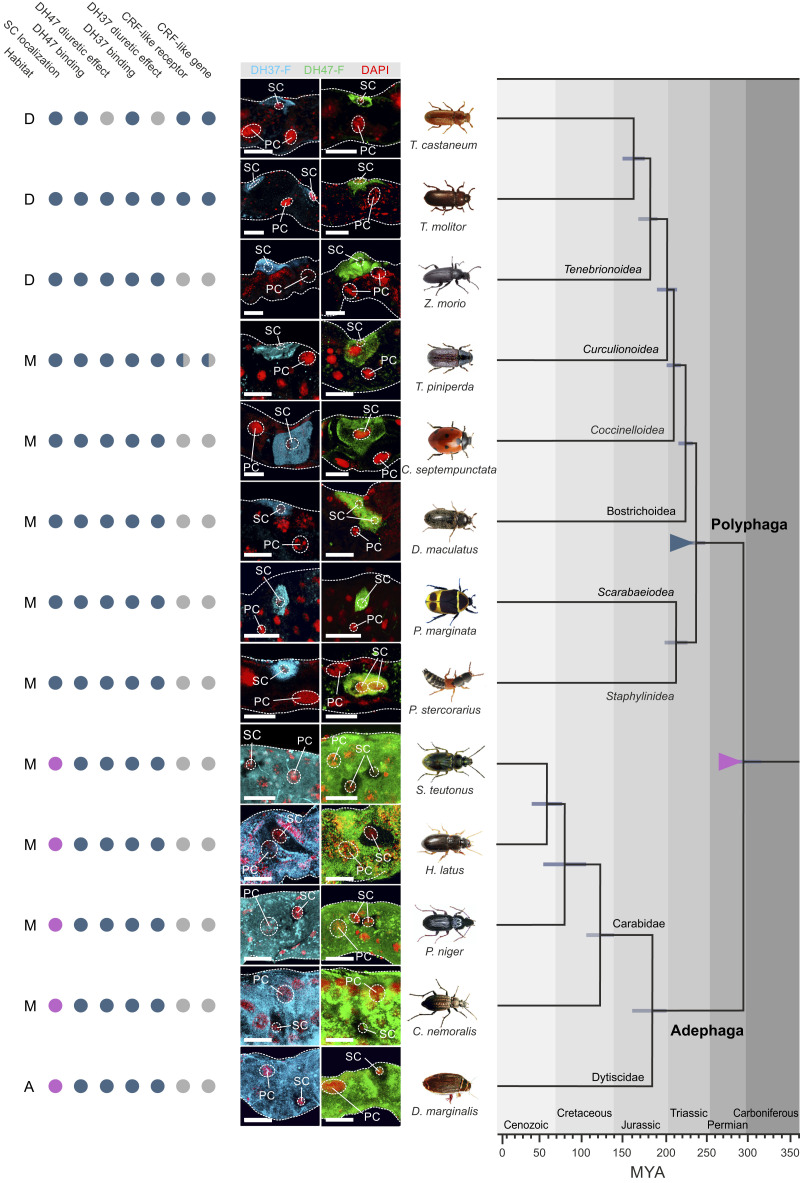
Mapping MT architecture across Coleoptera. Consensus phylogeny of the strategically chosen beetle species used (covering >70% of beetle biodiversity) in our study with superimposed character matrix. Closed blue circle denotes a positive; closed magenta indicates a negative for each category. Half-blue circles indicate that for members of that insect group a positive or negative has been experimentally confirmed. Gray closed circle implies that data are not available. The preferred environment of each species is denoted by: A, aquatic; D, dry; M, moist. Colored triangles indicate a significant event in beetle tubule function: magenta triangle; loss of Kinin signaling and emergence of alternative splicing of the CRF-like (*Urn8*) gene giving rise to two different ligands, DH37 and DH47. Blue triangle; SCs adopted Urn8 signaling to the exclusion of all cell types. The phylogenetic relationships and the horizontal bars representing 95% confidence intervals of divergence time for the branching nodes were adopted from Zhang et al. (15). (Scale bars, 20 μm.)

Collectively, our work points to a model in which beetle MT function operates via a two-cell–type model that is different from that of all other higher insects (11). To determine whether this epithelial organization is universal among beetles or, alternatively, a derived trait specific to the drought-resistant tenebrionid species, we adapted the ligand-receptor binding assay (as used in Fig. 2*C*) to systematically map MT tissue architecture across the beetle phylogeny. In our sampling strategy, we prioritized species representative of the larger beetle families while trying to select species inhabiting different types of ecological niches (dry, moist, or aquatic). This method allowed a compact sampling covering close to 300 million y of beetle evolution and more than 70% of beetle biodiversity (15). Using this approach, we detected specific and displaceable DH37-F and DH47-F binding to tubule basolateral membranes of all species sampled (Fig. 7), which is consistent with the ancestral origin of CRF-like signaling predating the radiation of the insects (42). Interestingly, although specific signals were detected in members of both the basal Adephaga and the more derived Polyphaga, the types of cells receiving the signals were different: In all polyphagan beetles, DH37-F and DH47-F binding localized entirely to a small-nucleated SC-type, whereas in the adephagan species peptide binding was confined to a larger PC-type (Fig. 7). These data suggest that the types of cells mediating neuropeptide action—although generally highly conserved across the insect phylogeny—have dramatically changed over the course of beetle evolution.

An implicit requirement of this approach is that DH37-F and DH47-F binding should predict a functional stimulation of beetle MT activity. We therefore adapted and optimized the Ramsay fluid secretion assay for all species studied, and measured the ability of both neuropeptides in stimulating fluid secretion. These results demonstrated that in every species in which specific DH37-F and DH47-F binding was observed, both peptides consistently stimulated diuresis with a trend toward DH37 being more potent in polyphagan beetles while the opposite was the case for DH47 in adephagan species (*SI Appendix*, Fig. S6).

Finally, to gain insight into the evolutionary origins of beetle tubule function and control we integrated all our experimental data with available genomic evidence and a consensus phylogeny of the investigated species of beetles (15). These results suggest that the loss of kinin signaling and the presence of an alternatively spliced *Urn8* gene resulting in two distinct DH hormones evolved early on in beetle evolution (magenta triangle, Fig. 7). Yet, more strikingly, our data reveal that a change in beetle MT architecture occurred ∼240 million years ago (Mya) in the last common ancestor of the Polyphaga suborders in which the smaller SC adopted Urn8 signaling to the exclusion of other cell types, a unique two-cell–type model (blue triangle, Fig. 7). Our data further indicate that beetle MT architecture is largely shaped by phylogeny and not environmental factors given that changes in tubule organization does not correlate with differences in habitat preference (dry, moist, or aquatic). Taken together, our results indicate that CRF-like signaling arose early in metazoan evolution, is universally diuretic in beetles, and underwent dramatic reorganization to be mediated by a functionally retooled SC that evolved alongside the diversification of the more advanced polyphagan suborders.

## Discussion

### A Two-Cell–Type Model Unique to Beetles.

The insect tubule is historically defined as the fastest secreting epithelium (per cell) in biology (43). It has since emerged that this ability depends critically on the separation of transport functions into distinct cell types, the classic two-cell–type model (1). Evidence suggests that this model is conserved across the higher insect Orders (3, 11), yet the secondary loss of kinin signaling (diagnostic of SC function outside Coleoptera) in beetles (12, 13) has raised the question: Do coleopterans entirely lack specialized SCs? In this study, we provide compelling evidence for the presence of a physiologically distinct SC in beetles, which has undergone dramatic molecular retooling to function in a fundamentally different way than SCs of other insects. Rather than mediating a kinin-stimulated chloride conductance (2), our data suggest that this functionally unique SC has adopted CRF-like Urn8 signaling from the larger PCs to regulate a privileged route for luminal directed K^+^ transport to control tubule secretion. These data imply that even though the differentiating characters of SCs are intrinsically linked with Tiptop expression, as in *D. melanogaster* (21), the gene regulatory network controlled by this important transcription factor has been reprogrammed in beetles. Yet, what is the functional significance of this modified tubule organization? One possibility is that the rapid colonization of osmotically hostile environments, in which beetles particularly thrive (44, 45), required adaptive changes in MT function to provide a tighter control of excretory water loss. Such a model is consistent with the secondary loss of the kinin pathway, as well as with potent antidiuretic effects of Capa and ADF signaling on tubule secretion (11, 19, 46), which collectively point to a general need to restrict diuretic activity and the associated fluid loss in beetles. Indeed, MT secretion in beetles has been suggested to predominantly act as a clearance mechanism in which the tubule fluid is recirculated by the rectal complex to concentrate metabolic waste in a way that does not affect the overall water balance of the insect (47). In *T. castaneum* and other drought-resistant beetle species, the distal ends of the MTs are closely applied to the rectum in a manner that creates a counter-current exchange system, or rectal complex, that is highly optimized to minimize water loss; in some species it even allows absorption of water vapor directly from moist air (30, 31). Even so, a role of DH37 and DH 47 as clearance hormones is not supported by our data, which clearly show that manipulating Urn8 signaling in vivo induces a marked diuretic effect that impacts whole-animal fluid balance. However, it is likely that the observed changes to tubule function and control are an integral part of the physiological adaptations that have allowed the higher polyphagan beetles to diversify and cope with some of the most challenging environments on the planet. It will be interesting to identify and characterize the molecular machinery responsible for mediating the Urn8-induced changes in tubule secretion to gain further insight into this two-cell–type model exclusively found in beetles.

### What the Brain Tells the Kidney.

A key requirement of homeostatic regulation is the ability to sense deviations in internal abundances and to initiate compensatory actions to restore balance. Our study shows that the *PI* neurons respond to the presence of low ECF osmolality by releasing DH37 and DH47 hormones into circulation, while high ECF osmolality conversely reduces their release and thus result in hormone accumulation. These data suggest that the *PI *neurons are sensitive to cues related to internal changes in osmolality and hereby act as a central command center for the control of systemic osmoregulation. Such a model implies that hemolymph changes are communicated across the blood–brain barrier to impact the *PI *neurons, which is supported by the high expression of aquaporins and other transport proteins in the glial cells of the insect blood–brain barrier (48). The mechanisms underpinning blood–brain barrier function, however, is limited and remains an important question for the future. While we do not know whether the *PI* neurons are directly osmosensitive, or alternatively if such information is encoded by other neurons, our data strongly suggest that this group of neurons communicates homeostatic needs for internal water in beetles. In *D. melanogaster*, the DH44 neurons of the *PI*, in addition to regulating tubule function (6), have been shown to act as postingestive nutrient sensors to help coordinate gut peristalsis and feeding (49, 50). Moreover, other subsets of neurons in the fly brain have been found to process information related to water and nutrient availability independent of gustatory sensory activation (51–54). These data imply that additional osmosensitive systems exist in *T. castaneum* and that Urn8 signaling may interact with other homeostatic programs to control both water and metabolic homeostasis. Indeed, it is likely that in the face of complex environmental challenges, multiple mechanisms converge to ensure a robust organismal response to diverse stressful conditions to sustain animal survival.

### Generality of a New MT Architecture.

Initially considered a trait unique to the large Order of Diptera, it is now becoming clear that a physiologically distinct SC mediating kinin action, chloride transport, and water flux represent an ancestral condition among the holometabolous insects (2, 3, 11). Although reports on deviations from this model exists within the Holometabola (55, 56), only beetles appear to lack such specialized SCs and therefore represent a striking exception to this generalized pattern (3, 11). Yet, by applying a modified ligand–receptor binding approach, we show that DH37 and DH47 reactivity maps exclusively to a new type of specialized SCs and that this cell type is present in all tested members of the large Polyphaga suborder. In contrast, in the more basal Adephaga, DH37 and DH47 selectively bind to the larger PCs consistent with the general pattern observed in all other higher insects (6, 23). These data imply that while CRF-like signaling evolved prior to the radiation of the insects (42), only the advanced polyphagan beetles possess a remodeled SC-type that has adopted Urn8 signaling to the exclusion of other cell types. Given that this unique MT architecture evolved at the same time as the diversification of the Polyphaga lineages, which contain nearly 90% of all extant species (15, 16), it is tempting to speculate that this tissue reorganization has conferred a selective advantage enabling beetles to become the most diverse and species-rich group on Earth.

**Fig. 8.**
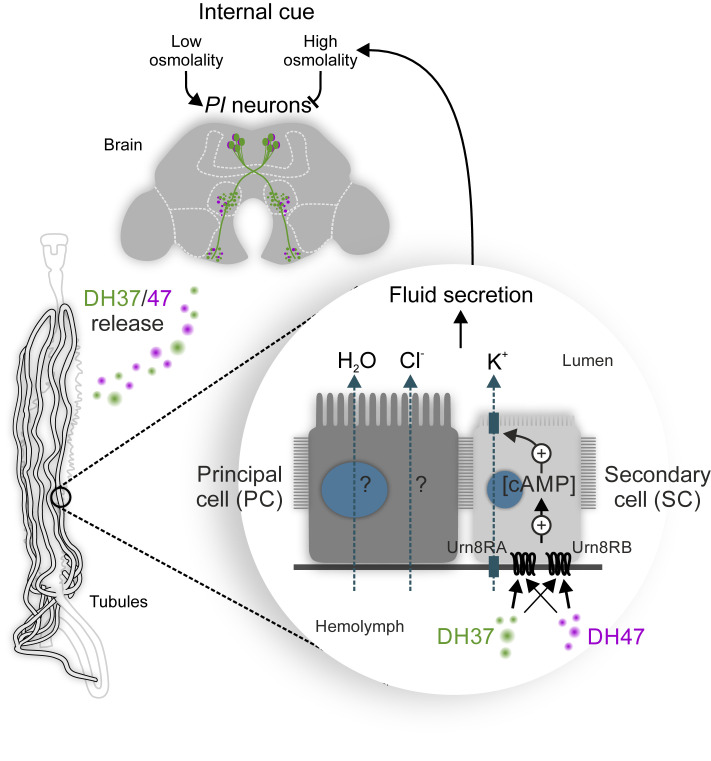
Model for the homeostatic control of systemic osmoregulation in *T. castaneum*. The brain *PI *neurons respond to internal changes in osmolality by releasing DH37 and DH47 hormones into the hemolymph to remotely activate Urn8R exclusively in SCs to increase K^+^ flux and stimulate tubule secretion via cAMP-dependent mechanism. The Urn8 circuit thus couples cues related to internal changes in water abundance to the homeostatic control of systemic osmoregulation in *T. castaneum* and perhaps in other higher beetle species.

In summary, our work uncovers a homeostatic program that is essential for the central control of systemic osmoregulation in beetles. This program operates via a subpopulation of osmosensitive neurons, which remotely control MT secretion via a two-cell–type model (Fig. 8) that evolved ∼240 Mya in the last common ancestor of the advanced Polyphaga beetles.

## Materials and Methods

### Animal Collections and Husbandry.

Developmentally synchronized *T. castaneum* (San Bernardino strain) stocks were maintained on organic wholemeal wheat flour supplemented with 5% (wt/wt) yeast powder (= *Tribolium* medium) at 30 °C at a constant 50% RH and 12:12 light:dark cycles, as in Halberg et al. (11). All other species used in this study were either commercially obtained or wild-caught, as described in the *SI Appendix*, *SI Materials and Methods*.

### Tissue Dissection and RNA Extraction.

Tissues were dissected and RNA was extracted from nonsedated sixth-instar larvae or 2-wk-old mature adults as described in *SI Appendix*, *SI Materials and Methods*.

### RNA-seq Analyses and Candidate GPCR Gene Filtering.

Total RNA libraries were sequenced on a BGISEQ-500 using paired-end chemistry and subsequent bioinformatic analyses were performed using the Tuxedo pipeline (57). To identify systemic signals that modulate MT activity, we prioritized GPCR genes with enriched expression in the MTs relative to the whole animal. For further information see *SI Appendix*, *SI Materials and Methods*.

### Molecular Cloning and Functional Characterization of Urn8 Receptor Isoforms.

cDNA of the *Urn8R* gene was synthesized from total RNA extracted from adult *T. castaneum* MTs and the coding regions of the two isoforms were amplified using isoform-specific primers (*SI Appendix*, Table S1). The PCR products were subsequently cloned into a vector and transfected into competent CHO/G16 cells to develop separate clone lines, which were subsequently used in a bioluminescence assay, as described in Egerod et al. (58). For further information see *SI Appendix*, *SI Materials and Methods*.

### Tissue-specific cAMP Detection.

MT cAMP production following ligand stimulation was measured using the time-resolved FRET (TR-FRET)-based LANCE ULTRA cAMP Kit (PerkinElmer) in combination with an EnSight Multimode Plate Reader (Perkin-Elmer) as described in *SI Appendix*, *SI Materials and Methods*.

### Peptide Synthesis.

Synthetic analogs of all peptides used were synthesized as described in *SI Appendix*, *SI Materials and Methods*.

### Generation of Antibodies and Visualization of Ligands and Receptor Distribution.

To generate anti-Urn8R, anti-DH37 and anti-DH47 specific antibodies, we analyzed the amino acid sequence of the proteins to identify the most optimal immunizing peptide region according to a previously described method (59). These antibodies were subsequently used for immunocytochemistry (60) to visualize immunofluoresence in different tissues. Where necessary, immunofluorescence levels were quantified using the FIJI software package from images acquired using identical microscope settings as described in Texada et al. (61). For further information see *SI Appendix*, *SI Materials and Methods*.

### Environmental Stress Exposure.

Beetles were subjected to different environmental stressors as described in *SI Appendix*, *SI Materials and Methods*.

### Hemolymph Collection and Quantification.

Hemolymph was collected according to a modified protocol (62) from animals exposed to the different environmental stress exposures. See *SI Appendix*, *SI Materials and Methods* for further information.

### Ex Vivo Organ Culture.

Organ culture experiments and DH37 and DH47 retention levels were measured as described in *SI Appendix*, *SI Materials and Methods*.

### ELISA Detection of Circulating DH37 Levels.

Hemolymph was collected from adult *T. molitor* exposed to either high or low humidity conditions for 7 d, and the circulating DH37 protein levels were quantified using an ELISA (61), as described in *SI Appendix*, *SI Materials and Methods*.

### Ramsay Fluid Secretion Assay.

Assays were carried out on intact live tubules as described in *SI Appendix*, *SI Materials and Methods*.

### Ligand–Receptor Binding Assay.

The ex vivo receptor-binding assay was performed on live intact tubules from different species as described in refs. 11, 22, and 23. For further details, see *SI Appendix*, *SI Materials and Methods*.

### Electrophysiological Assays.

SIET and TEP measurements were performed on free isolated MTs from *T. molitor*, as described in detail in refs. 29 and 55. See *SI Appendix*, *SI Materials and Methods* for further information.

### Production of Double-Stranded RNA and RNAi-Mediated Knockdown.

Double-stranded RNA (dsRNA) synthesis and knockdown of target gene expression by RNAi was carried out according to a protocol described in *SI Appendix*, *SI Materials and Methods*.

### Gene-Expression Analysis.

Validation of RNAi-mediated gene knockdown and environmentally induced changes in gene expression (*SI Appendix*, Table S1) was assessed by qRT-PCR, as described in *SI Appendix*, *SI Materials and Methods*.

### Quantification of Water Content.

Gravimetric estimates of body water were made by measuring wet and dry body weight after desiccation in control and *Urn8R* knockdown animals; see *SI Appendix*, *SI Materials and Methods* for further details.

### Defecation Behavior.

The effects of manipulating Urn8-signaling on in vivo whole-animal excretory behavior was performed as described in *SI Appendix*, *SI Materials and Methods*.

### Statistics.

Data analysis was performed for each experimental condition using relevant methods as described in *SI Appendix*, *SI Materials and Methods*.

## Supplementary Material

Supplementary File

## Data Availability

All study data are included in the article and *SI Appendix*.
