# Supplementary material for: A unique Malpighian tubule architecture in Tribolium castaneum informs the evolutionary origins of systemic osmoregulation in beetles
Source: Proc Natl Acad Sci U S A. 2021 Mar 30;118(14):e2023314118. doi: 10.1073/pnas.2023314118 (PMC8040626; doi:10.1073/pnas.2023314118)
Supplement: Supplementary File [file pnas.2023314118.sapp.pdf]

PNAS
www.pnas.org

Supplementary Information for

A unique Malpighian tubule architecture in *Tribolium castaneum* informs the evolutionary origins of systemic osmoregulation in Beetles

Takashi Koyama[a], Muhammad Tayyib Naseem[a], Dennis Kolosov[b,d], Camilla Trang Vo[a], Duncan Mahon[a], Amanda Sofie Seger Jakobsen[a], Rasmus Lycke Jensen[a], Barry Denholm[c], Michael O'Donnell[b] and Kenneth Veland Halberg[a,*]

[a] Department of Biology, University of Copenhagen, DK-2100 Copenhagen, Denmark
[b] Department of Biology, McMaster University, Ontario, Canada L8S 4K1
[c] Centre for Discovery Brain Sciences, University of Edinburgh, Edinburgh EH8 9AG, UK
[d] Department of Biological Sciences, California State University San Marcos, CA 92069, US

* Kenneth Veland Halberg, Universitetsparken 15, Building 3, 3rd floor, DK-2100 Copenhagen, Denmark, phone number: +45 26810458
Email: kahalberg@bio.ku.dk

This PDF file includes:

SI Materials and Methods
Figures S1 to S6
Tables S1
SI References

SI Materials and Methods

Animal Collections and Husbandry. Developmentally synchronized *Tribolium castaneum* (San Bernardino strain) stocks were maintained on organic wholemeal wheat flour supplemented with 5% (w/w) yeast powder (= *Tribolium* medium) at 30°C at a constant 50% RH and 12:12 light-dark cycles as in (1). *Tenebrio molitor* and *Zophobas morio* were both cultured on organic bran supplemented with occasional potato slices under identical environmental conditions. *Tomicus piniperda* were kept in the Pine tree bark they were collected from, and were kindly gifted by Prof. Lawrence Kirkendall, Bergen University, NO. *Pacnoda marginata* was acquired from Insektorama, DK, and subsequently kept on pieces of rotten fruit and detritus. *Dermestes maculatus* were sourced commercially from Fera Science Ltd, UK, and were reared on a diet of fishmeal and yeast (16:1) supplemented with bacon. Specimens of *Coccinella septumpunctata, Platydracus stercorarius, Stenolophus teutonus, Harpalus latus, Pterostichus niger, Carabus nemoralis* and *Dytiscus marginalis* were all wild-caught at their respective habitats in DK, and immediately used for experimentation.

Tissue Dissection and RNA Extraction. Tissues were dissected from non-sedated 6th instar larvae or 2-week-old mature adults under a freshly prepared mixture of Schneider's medium and *Tribolium castaneum* saline (1:1, v/v). The *T. castaneum* saline contained: NaCl 90 mmol L^{-1}, KCl 50 mmol L^{-1}, $MgCl_2$ 5 mmol L^{-1}, $CaCl_2$ 2 mmol L^{-1}, $NaHCO_3$ 6 mmol L^{-1}, NaH_2PO_4 6 mmol L^{-1}, Glucose 50 mmol L^{-1} and the pH was adjusted to 7.0. Dissected tissues were then transferred to 500 µl QIAzol (Qiagen, Hilden, DE) and stored at -80°C until sufficient tissue had been collected to allow extraction of a minimum of 200 ng RNA in total. Next, the samples were thawed and physically disrupted using a beadmill (1 min max speed) using a TissueLyser LT (Qiagen, Hilden, DE) and then exposed to an in-house phenol-chloroform extraction protocol including an extra chloroform step and several RNA washing steps. The RNA was then finally purified using a Qiagen RNeasy Plus mini kit according to the manufacturer's instructions. The optional DNase step, the optional drying of the column, and back-elution were all included. For each sample, the concentration of RNA was determined using a NanoDrop 1000 Spectrophotometer (ThermoFisher, MA, USA), and the quality of the RNA was determined using an Experion Pro260 (Bio-Rad, CA, USA) according to the manufacturer's instructions. Each tissue sample was prepared in biological triplicates.

RNA-seq Analyses and Candidate GPCR Gene Filtering. Total RNA libraries were prepared for each sample according to a low-input protocol by BGI Genomics (Shenzhen, Guangdong, China), and sequenced on a BGISEQ-500 using paired-end chemistry (100 nt reads) with a sequencing depth of 6 Gb per sample (i.e. approaching 40x of the ~150 Mb *Tribolium castaneum* genome). The subsequent bioinformatic analyses were performed using the Tuxedo pipeline (2), and the latest version for the *T. castaneum* reference genome (3). The full data sets were used to populate a database, BeetleAtlas, which constitutes a tissue-specific transcriptomic atlas for *T. castaneum* that will be made publically available in a separate publication. Of the known neuropeptide receptor genes, members of G-protein coupled receptor (GPCR) family are obvious candidates (4). Accordingly, we interrogated BeetleAtlas for all GPCR genes with significant expression in the Malpighian tubules, and prioritized these according to signal FPKM (Fragments Per Kilobase of transcript per Million mapped reads) intensity, to help identify systemic signals that modulate MT activity. Of these, the gene *TC034462* – encoding a CRF-like receptor we have named Urn8R – shows the highest expression, and a full tissue-specific expression analysis of all predicted isoforms of this gene was performed.

Molecular Cloning and Functional Characterization of Urn8 Receptor isoforms.
The *Urn8R* gene is expressed in three isoforms, but only RA (1359bp) and RB (1446bp) are predicted to possess the stereotypic 7-transmembrane structure of GPCRs and therefore the only ones selected for cloning. cDNA was synthesized from total RNA extracted from adult *T. castaneum* MTs using the High-Capacity cDNA Reverse Transcription Kit with RNase Inhibitor (ThermoFisher, MA, USA), and the coding regions of the two isoforms were amplified using Q5® Hot Start High-Fidelity 2X Master Mix (New England Biolabs, MA, USA) using isoform-specific primers (Supplemental Table 1). The PCR products were subsequently cloned into an EcoRI-

HF® (New England BioLab, MA, USA) linearized pIRESZ2_ZsGreen1 vector using In-Fusion® HD cloning kit (TaKaRa Bio Inc, Kusatsu, JP). After sequence validation (Eurofin, Luxemburg, LU), these plasmids were transfected into competent CHO/G16 cells to develop separate stable clone lines, which were subsequently used in a bioluminescence assay as described in (5).

Tissue-specific cAMP detection. MT cAMP production following ligand stimulation was measured using the time-resolved fluorescence energy transfer (TR-FRET) based LANCE ULTRA cAMP Kit (PerkinElmer, MA, USA) in combination with an EnSight Multimode Plate Reader (Perkin Elmer, MA., USA). In brief, whole MTs were acutely dissected from adult *T. castaneum* as described above. Then, exactly 10 full-length MTs in stimulation buffer (control), or stimulation buffer supplemented with either DH37 or DH47 at a concentration ranging from 10^{-13} M to 10^{-6} M, were transferred to individual wells on an OptiPlate-384 (Perkin Elmer, MA., USA). Each sample concentration was setup in 3-6 biological replicates. The loaded plate was then left to incubate at room temperature for 30 min, before adding 5μL 4X EU-cAMP tracer and 5μL of 4X Ulight-anti-cAMP working solutions. The plate was then left to incubate with TopSeal-A sealing film for 1 h, before being measured on the EnSight Multimode Plate Reader, using the TR-FRET program. To calculate absolute changes in cAMP production, we additionally constructed a standard curve, which allowed us to plot a dose-response curve in nM cAMP/tubule.

Peptide Synthesis. Synthetic analogues of all peptides used were synthesized by Cambridge Peptides (Birmingham, UK) at a purity of >90%. For *T. castaneum* DH37 and DH47 ligands, versions with an N-terminal cysteine were additionally made in order to conjugate a TMR-C_5-maleimide Bodipy dye (BioRad, CA, USA), to make fluorescent TMR-C_5-maleimide-SPTISITAPIDVLRKTWAKENMRKQMQINREYLKNLQamide (DH37-F) and TMR-C_5-maleimide-AGALGESGASLSIVNSLDVLRNRLLLEIARKKAKEGANRNRQILLSLamide (DH47-F). All peptide concentrations were corrected according to peptide purity.

Generation of Antibodies and visualization of Ligands and Receptor distribution. To generate antibodies specific against proteins of interest, we analyzed the amino acid (aa) sequence of the proteins to identify the most optimal immunizing peptide region according to a previously described method (6). For Urn8R, this analysis resulted in the selection of a peptide corresponding to aa 3–17 (WSEPLPQEPEPVDAD) in the N-terminal region of the full-length parent protein, which was then submitted for a custom immunization protocol carried out by Genosphere Biotechnologies (Paris, France). Additionally, aa 9-23 (PIDVLRKTWAKENMRK) and aa 27-41 (IARKKAKEGANRNRQILLSL) of the mature DH37 and DH47 peptides, respectively, were also selected for preparation of polyclona antisera. Epitope specificity of the different antisera was established by comparing wild type and RNAi animals by immunostaining as well as by co-application of the pre-immune serum. Additionally, the specificity of the anti-Urn8R antiserum was tested by western blotting. Dissected MTs were lysed in 50ul of RIPA buffer (25mM Tris, 150mM NaCl, 0.5% sodium deoxycholate, 1% Triton X-100) + Halt protease and phosphatase inhibitor cocktail 100X (100:1; ThermoFisher, MO, USA), and homogenized using a beadmill. Next, the sample was centrifuged at 14,000 x *g* at 4°C for 15 min to pellet debris, before adding x2 Laemmli buffer (Bio-Rad, CA, USA) in a ratio of 1:1 and heat-treating at 95°C for 5 min. The sample was then electrophoresed through a 4-20% precast polyacrylamide gradient gel (Bio-Rad, CA, USA). Proteins were transferred to polyvinylidene difluoride membrane (Millipore, MA, USA), and the membrane was blocked with Odyssey Blocking Buffer (LI-COR, NE, USA). Next, the membrane was incubated with rabbit anti-Urn8R (1:1000) and mouse anti-tubulin (1:2500; Sigma-Alrich, MO, USA) in blocking buffer supplemented with 0.2% Tween 20 (w/v). Primary antisera were detected with goat secondary antibodies – IRDye 680RD anti-mouse and IRDye 800CW anti-rabbit diluted (1:10000) – and bands visualized using an Odyssey Fc imaging system.

Immunocytochemistry (ICC) was performed as in (7). In brief, appropriate tissues were dissected and fixed in 4% paraformaldehyde in PBS for 20 min. Tissues were then washed four-six times in PBST (PBS + 0.1% Triton X-100), blocked with PBST containing 3 % normal goat serum (blockPBST; Sigma-Aldrich, MO, USA) for 1 h, and incubated in primary antibodies. Primary antibodies used were polyclonal rabbit anti-Urn8R (1:200), polyclonal rat α-DH37 (1:500)

and polyclonal guinea pig α-DH47 (1:500). The subcellular localization of the endogenous proteins were visualized by applying Alexa Fluor 488/555/647anti-rabbit, anti-rat or anti-guinea pig secondary antibodies (1:500; Sigma Aldrich, MO, USA) in combination with DAPI (1:1000) and Rhodamine-conjugated Phalloidin (1:500; Sigma Aldrich, MO, USA) in blockPBST overnight at 4°C. Following several washes, first in PBST and then in PBS, the different tissues were mounted on poly-L-lysine coated dishes 35mm glass bottom dishes (MatTek Corporation, MA, USA) in Vectashield (Vector Laboratories Inc., CA, USA) and imaged on an inverted Zeiss LSM800 confocal microscope equipped with airy scan technology (Zeiss, Oberkochen, DE). Where necessary, immunofluorescence levels were quantified using the FIJI software package from images acquired using identical microscope settings as described in (8)

Environmental stress exposure. In fed (control) conditions, animals were housed individually in 96-well plate with standard *Tribolium* medium as described above. For drinking only (water) treatments, animals were kept in 96-well plates with a small block of 1% agar with 0.05% bromophenol blue (BPB); drinking was verified by the presence of blue deposits. For desiccation treatments, animals were kept in 96-well plates with a piece of filter paper without any nutritional and water sources with individual plates being kept at 5, 50 or 90% RH, respectively, at 30ºC.

Hemolymph collection and quantification. Hemolymph was collected according to a modified protocol (9) from animals exposed to the different environmental stress exposures as described above. In brief, animals were washed and subsequently dried on tissue paper for 2 hours to remove moisture. Then, beetles were anesthetized by CO_2 and their cuticle pierced between the pronotum and elytron before being transferred to an ice-cold 0.5-ml tube with a small hole in the bottom in groups of 10. This tube was then placed in a larger 1.5-ml collecting tube, which was centrifuged at 12,000 x *g* for 15 min at 4°C. Hemolymph from three separate tubes were combined into each collecting tubes (containing 500 µl paraffin oil to prevent oxygen-induced melanization) from each environmental condition. Following sample collection, each sample was diluted to a final volume of 50 µl with ddH2O and the osmotic pressure of each sample was measured in triplicates on VAPRO Vapor Pressure Osmometer Model 5600 (Wescor Inc., UT, USA) with each measurement corrected according to the dilution factor of the sample.

***Ex-vivo* organ culture.** For organ culture experiments, brains were dissected from 2-week old mature adults in cold *Tribolium* saline. Brains were then divided into groups of 8-10 brains, and each group incubated in 500 µL *Tribolium* saline of different osmotic strengths (-200, -100, 0 or +200mOsm) containing 5% feta bovine serum (Sigma-Aldrich, MO, USA) prepared according to a previously described protocol (10). The samples were incubated for different durations (0 h, 1 h or 4 h) in humidity chambers at room temperature. After the respective incubation periods, the brains were removed for ICC, and the DH37 and DH47 retention levels were measured as described above.

ELISA detection of circulating DH37 levels. To detect circulating DH37 peptide levels, hemolymph was collected from adult *T. molitor* exposed to either high (RH 90%) or low (RH 5%) humidity conditions for a period of 7 days. In brief, animals were anesthetized by CO_2 and then pierced on the dorsal side as described above, before being gently squeezed to collect the clear hemolymph using a p20 micropipette. The collected hemolymph was then immediately transferred to a pre-chilled collection tube containing a small amount of N-Phenylthiourea (to prevent hemolymph melanization) and placed on dry ice, with hemolymph pooled from 5-10 animals to achieve approximately 50 µl per sample; a minimum of 5 samples were collected in total for each condition. Next, the samples were heat in-activated at 60°C for 5 min and subsequently centrifuged at 2,000×*g* for 2 min at 4 °C. The supernatant was transferred to a new tube and stored at -80°C until further processing. Following sample collection, the DH37 levels in hemolymph was quantified using a modified ELISA protocol (8). Briefly, wells of a 96-well plate was coated using a polyclonal rat α-DH37 (1:20) diluted in coating buffer (2.12 g of Na_2CO_3 and 6.72 g of $NaHCO_3$ in 500 ml of ddH_2O) overnight at 4°C. Next, the wells were rinsed 5 times in PBT and blocked for 1 hour in blocking buffer (PBT + 4% milk powder). Hemolymph samples were then diluted (3:2) in blocking buffer and 100 µl of sample (N=4) and standards (duplicates of 10^{-7}M to 10^{-9}M of DH37 peptide)

were pipetted into appropriate wells and incubated overnight at 4°C on gentle shaking. Following several washes in PBT, a 100 µl of detection solution containing rabbit α-DH37 (11) in blocking buffer (1:1000: generous gift from Dr. Liliane Schoofs, KU Leuven, Belgium) was added to each well and incubated for 2 hours at room temperature. Each well was then washed several times in PBT and then incubated in HRP-conjugated anti-rabbit IgG (1:20000; SigmaAldrich) in blocking buffer on gentle shaking. The solution was then aspirated and the wells washed a final time. Finally, each well was added a 100 µl of TMB substrate solution (SigmaAldrich) and incubated for 30 min, before being added 100 µl stop solution (2M sulfuric acid). The absorbance of each well was measured at 450 nm on an EnSight Multimode Plate Reader Ensight, and the amount of DH37 in each sample was then calculated from the standard curve.

Ramsay Fluid Secretion Assay. Fluid secretions were measured according to a modified version of the method described in (1). In brief, intact MTs were carefully dissected from whole animals and set-up as in vitro preparations by isolating them in drops of Schneider's medium and *Tribolium* saline (1:1, v/v) under water-saturated liquid paraffin oil, with both ends wrapped around two oppositely placed minuten pins and the middle region bathed in the saline. Next, a small hole was introduced mid-way between the saline drop and the pin, thereby allowing the secreted fluid to accumulate as a discrete droplet. The volume of the secreted fluid was then collected at distinct time intervals and the volume calculated according to $(4/3)\pi r^3$ by measuring the diameter of the droplet using an ocular micrometer. An increase in fluid secretion rate following DH37 or DH47 application compared to unstimulated basal conditions was taken as an indication of a diuretic effect. For each species, the above-mentioned protocol was modified to accommodate the vast difference in size and function of the tubules.

Ligand-Receptor Binding Assay. The *ex vivo* receptor-binding assay was performed as described in (1, 12, 13). Tubules were carefully dissected from specimens of each species under Schneider's and *Tribolium* saline and then mounted on poly-L-lysine-covered 35mm glass bottom dishes. Next, the tissues were set-up in a matched-pair protocol, in which one batch was incubated in the appropriate insect saline added the labelled neuropeptide analogue (10^{-6} M) and DAPI (1 µg ml^{-1}), while the other was incubated in just DAPI; the latter batch was used to adjust baseline filter and exposure settings to minimize auto-fluorescence during image acquisition. Images were subsequently recorded on a Zeiss LSM 800 confocal microscope using baseline filter and exposure settings. A concentration of 10^{-6}M of the peptide analogues was chosen for assay, as this was shown to be the minimal concentration needed to produce a saturated receptor response, thereby optimizing the conditions for optical detection of ligand–receptor complexes. Competitive displacement of the labelled ligand under identical microscope settings, following co-application of the labelled (10^{-7}M) and unlabeled (10^{-5}M) ligands was taken as an indication of binding specificity.

Electrophysiological assays. Scanning Ion-selective Electrode Technique (SIET) and transepithelial potential (TEP) measurements were performed on free isolated MTs from *Tenebrio molitor* as described in detail in (14, 15). In brief, the ion-selective microelectrode voltage was measured at a position close to the tissue (3–5 µm) and subsequently at a more distant position (app. 50 µm) from the tubule. The mean measured voltage difference for three replicate measurements between the inner and outer limits of excursion was converted into a concentration difference. Ion flux was estimated from the measured concentration difference using Fick's law. For TEP measurements, isolated MTs were transferred immediately after dissection to a poly-L-lysine-coated Petri dish filled with *Tribolium* saline. TEP was measured by impaling the tubule lumen with a sharp microelectrode pulled from double-barreled theta-glass (World Precision Instruments, Inc. FL, USA) with reference to the basolateral bath. The TEP depolarization before and after neuropeptide treatment (10^{-7}M) was recorded with a high impedance dual channel differential electrometer HiZ-223 (Warner Instruments, CT, USA) that was connected to PowerLab data acquisition system running LabChart software (ADI Instruments, Oxford, UK).

Production of dsRNA and RNAi-mediated Knockdown. To silence target gene expression by RNAi, transcript sequences covering app. 200-500 bp were selected. Total RNA was then extracted from either tubules or heads (showing highest enrichment of *Urn8R* or *Urn8*, respectively) and cDNA synthesis were carried out as described above. Using the cDNA as template, fragments were amplified by PCR using gene-specific primers that were tagged with T7 promoter sequences in both 3-prime and 5-prime ends (see Supplemental Table 1). These gene specific fragments were then cloned into the pUC19 vector individually and subsequently verified by sequencing (Eurofins, Luxembourg, L). Using the cloned vector as template, bidirectional *in vitro* transcription was carried out using the MEGAscript T7 transcription kit (ThermoFishcer, MA, USA), and the quality of the resulting dsRNA was checked by gel electrophoresis and quantified using NanoDrop. The concentration was adjusted to 2 µg/ul using injection buffer (1.4 mM NaCl, 0.07 mM Na_2HPO_4, 0.03 mM KH_2PO_4, 4 mM KCl), and a total of 500 nl dsRNA solution was injected into age-matched adults using a Nanoject II injector (Drummond Scientific, PA, USA). Animals were allowed to recover for 3 days post-injection before used for experimentation.

Gene expression analysis. Validation of RNAi-mediated gene knockdown and environmentally induced changes in gene expression was assessed by quantitative Real-Time PCR (qPCR). Total RNA extraction was carried out 3 days post dsRNA injection and cDNA synthesis were carried out as described above. Next, qPCR was performed using the QuantiTect SYBR Green PCR Kit (Fisher Scientific, NH, USA) in combination with a Stratagene Mx3005P qPCR system (Agilent Technologies, CA, USA). Expression levels were normalized against the housekeeping gene *rp49*. All primers used are listed in Supplemental Table 1.

Desiccation tolerance. Animals were kept on *Tribolium* medium for 3 days after dsRNA injection. Healthy animals were then transferred to a 96-well plate in a container filled with silica gel beads (Sigma-Aldrich, MO, USA) to produce a low humidity environment (app. RH 5% - measured by a custom-build hygrometer). The number of dead animals (not responding to tactile stimuli) were then counted every 4-8 h for 7 days. Data were expressed as percent survival over time.

Quantification of water content. To measure changes in total water content, individual beetles were transferred to a small plastic container and then measured on a Sartorius SE2 ultra micro balance (= W_T; Sartorius, Göttingen, DE; 0.1 µg readability). The animals were then housed under low humidity conditions as described above, and after 48 h the beetles were reweighted (= W_{48}). To measure the corresponding dry weight of the animals, they were kept at -20°C over night and then placed in a 65°C incubator for at least 2 days before being weighed a final time (=W_{dry}). The percent water loss of total body water for each animal was calculated as $(W_T – W_{T48})/(W_T – W_{dry}) \times 100\%$, with *N*=29 animals weighed for each experimental group.

Defecation Behavior. To assess the effects of manipulating Urn8-signalling on whole-animal excretory behavior *in vivo*, dsRNA-injected animals were starved for 2 days followed by refeeding a standard *Tribolium* medium supplemented with 0.05% (w/w) Bromophenol blue (BPB) sodium salt (Sigma-Aldrich, MO, USA) overnight. This special medium was created by mixing the standard *Tribolium* medium with BPB and a small amount of water hereby creating a uniform paste, which was left to dry at room temperature overnight. The dried BPB-labelled *Tribolium* medium was then ground to a fine powder creating a consistency identical to that of the standard medium. Beetles were then placed in individual wells of a 96-well plate fitted with a small piece of filter paper and the number of BPB-labelled deposits produced by each animal over a 4 h period was quantified. The same approach was used to test the physiological effects of DH37 or DH47 hormone stimulation on *in vivo* excretion, by injecting groups of animals with either PBS or PBS containing DH37 or DH47 peptide corresponding to a final peptide concentration of app. 10^{-7}M. A minimum of 18-37 animals was used in each experimental group.

Statistics. The statistical analyses were performed using the data analysis software GraphPad Prism 8 (CA, USA). The normal (Gaussian) distribution of data were tested using D-Agostino-Pearsen omnibus normality test. Data are plotted as mean ± SEM, Tukey's box-and-whisker plots

or as violin plots as indicated in each figure legend. Statistical differences between one control group and another group (unpaired samples) or between the same groups at different time points (paired samples) were compared using two-tailed Student *t*-test, whereas differences between one control group and several other groups were pairwise compared by one-way ANOVA followed by Dunnett's multiple comparisons tests taking P=0.05 (two-tailed) as the critical value. P-values are indicated as: * P < 0.05, ** P < 0.01, *** P < 0.001, **** P < 0.0001.

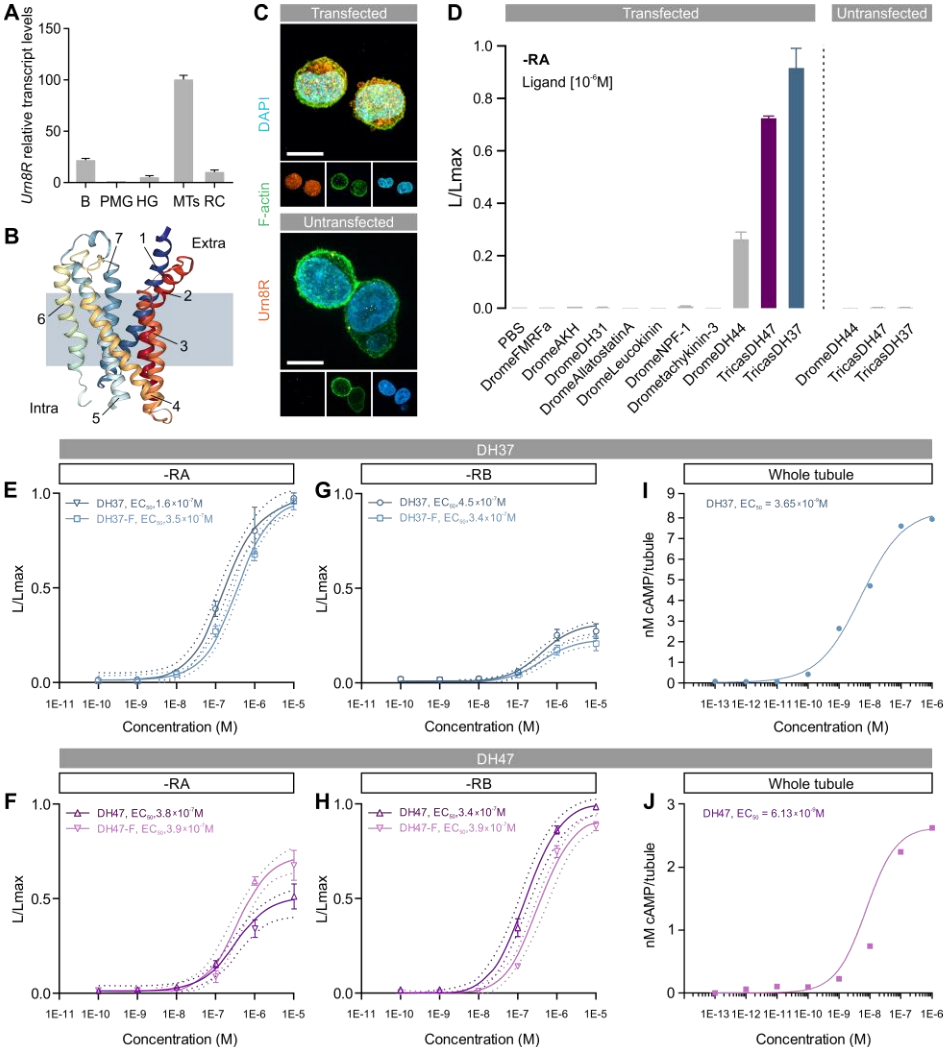

Fig. S1. Deorphanization and functional characterization of the Urn8 receptor. *(A)* Validation of *Urn8R* expression by qPCR across selected tissues. Brain, B; Posterior midgut, PMG; Hindgut, HG; Malpighian tubules, MTs; Rectal complex, RC. *(B)* Three-dimensional homology modeling of Urn8R shows that the receptor possesses the stereotypic seven-transmembrane structure of GPCRs, and that it belongs to the CRF-like family of receptors in the Class B secretin-like subfamily of GPCRs. *(C)* Verification of *Urn8R* transfection into competent CHO/G-16 cells using a custom-made rabbit-anti-Urn8R antibody. Specific immunoreactivity at the cell membrane was only detected in cells transfected with cDNA coding for the Urn8R indicating that the receptor is properly expressed and trafficked. *(D)* Bioluminescence responses of stably transfected cells (-RA isoform) following addition of different peptide analogues at a concentration of 10^{-6}M. *D. melanogaster* CRF-like ligand (DromeDH44) induces partial activation while the putative *T. castaneum* CRF-like agonists (tricasDH37 and tricasDH47) induce a larger receptor activation. No response was detected in untransfected cells. *(E-H)* Dose-response curves of isoform -RA (E-F) and -RB (G-H) following application of fluorophore-coupled and uncoupled DH37 and DH47. Isoform -RA shows stronger activation by DH37 compared to DH47, which only induces a partial receptor response. Isoform -RB shows high activation by DH47, and a pronounced smaller receptor activity following DH37 stimulation. Both labelled and unlabeled peptides induce similar receptor responses with comparable potencies. *(I-J)* Dose-response curves of cAMP production as measured by the ultra-sensitive FRET-based LANCE ULTRA method in MTs stimulated with either DH37 or DH47 peptide.

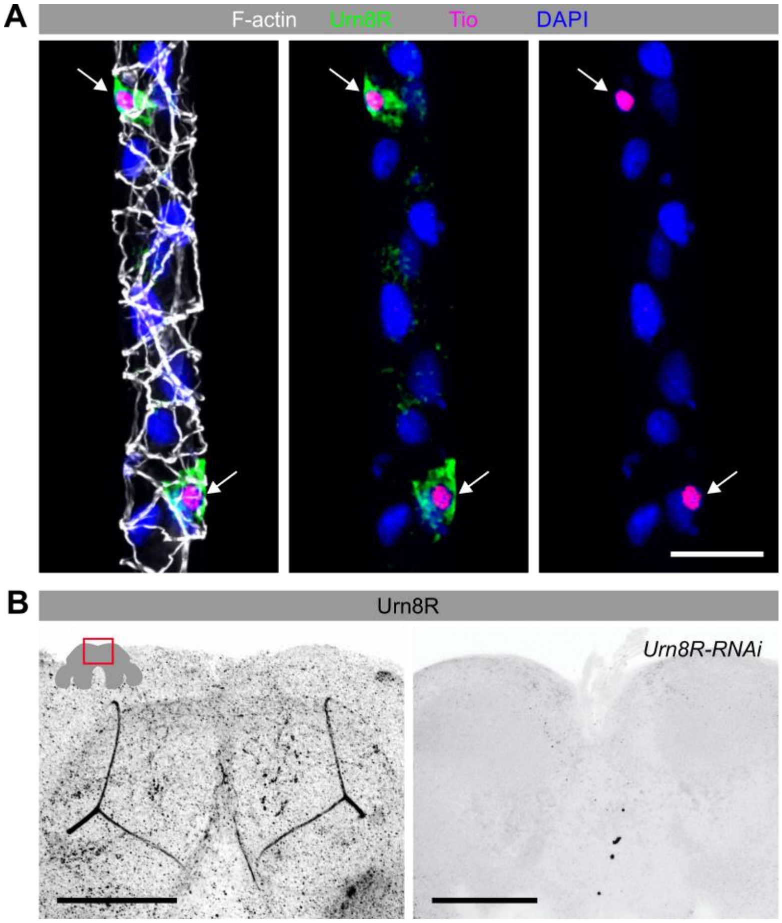

Fig. S2. Validation of Urn8R expression. (*A*) The Tiptop (Tio) transcription factor involved in SC differentiation in *D. melanogaster* and other insects co-localize with Urn8R in all SCs in *T. castaneum* tubules. (*B*) Urn8R is also expressed in the adult *T. castaneum* brain in a region called the mushroom body. The immunoreactivity disappears in brains dissected from animals in which *Urn8R* had been knocked down (*Urn8R-RNAi*).

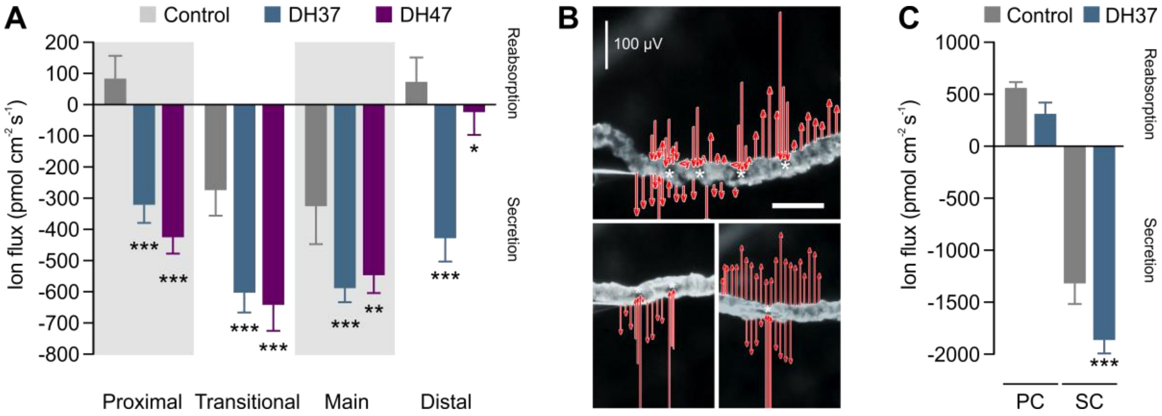

Fig. S3. Regional and cell-specific effects of DH37 and DH47 on K^+ secretion in four different functional regions of the tubule as measured by SIET. *(A)* Both DH37 and DH47 increase net K^+ secretion in all four regions of the tubule, but DH37 appears significantly more potent than DH47 in the distal region. All data are presented as mean ± SEM. (one-way ANOVA; * $P < 0.05$, ** $P < 0.01$, *** $P < 0.001$; n=6). *(B)* Potassium conductance hot spots colocalizes with the anatomical position of secondary cells (asterisks). Arrow length indicates the magnitude of potassium flux. Scale bar = 70µm. (*C*) Changes in potassium ion flux before and after DH37 stimulation in PCs and SCs, respectively (Student's *t*-test; *** $P < 0.001$; n=3).

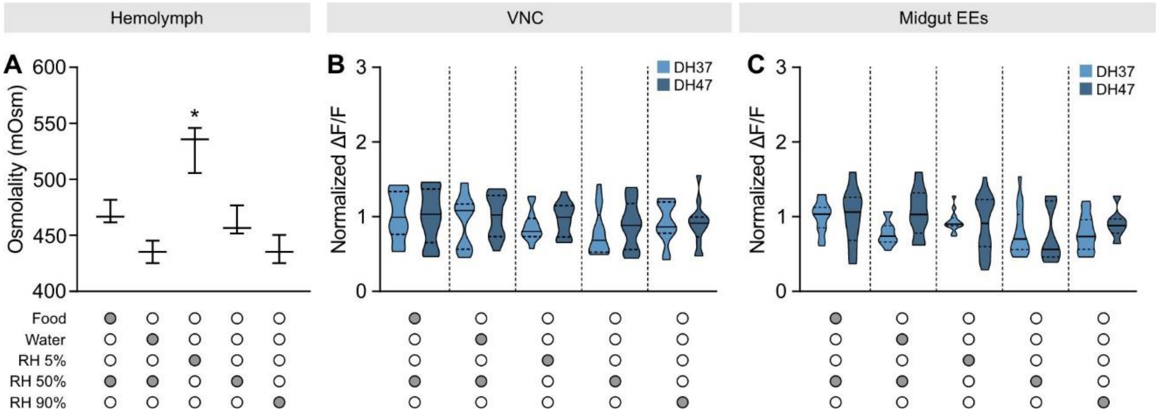

Fig S4. Changes in hemolymph osmotic pressure and DH37/DH47 release during different environmental exposures. (*A*) Hemolymph osmolality measured from animals exposed to different environmental exposures (one-way ANOVA; * $P < 0.05$; n=3). (*B*-*C*) Violin plots of intracellular DH37 and DH47 peptide levels from ventral nerve cord (VNC) and midgut enteroendocrine cells (EE) from animals exposed to different environmental conditions (one-way ANOVA; n=15-27).

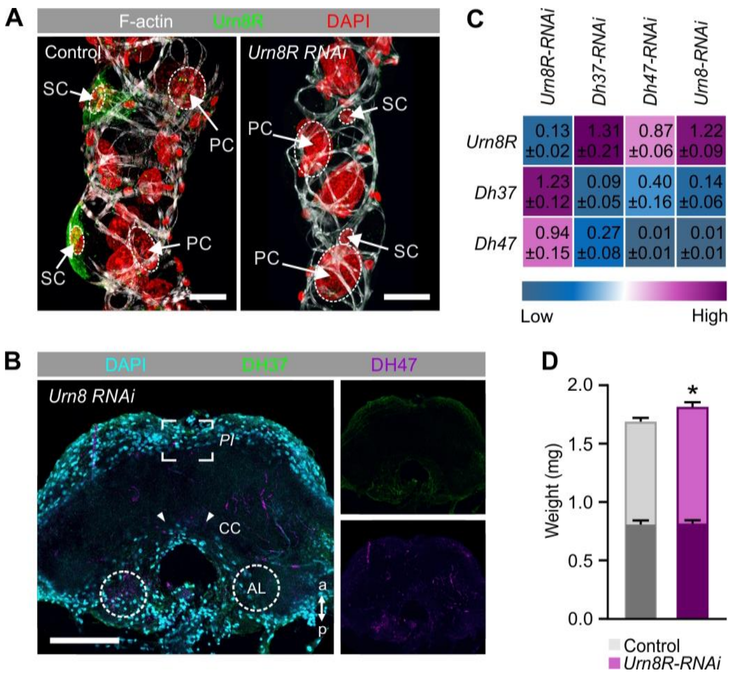

Fig S5. Validation of Urn8/Urn8R knockdown efficacy. (*A*) *T. castaneum* wild-type and *Urn8R* knockdown MTs showing Urn8R expression (green) counterstained with F-actin (white) and DAPI (red) in maximum projected z-stack. Scale bar = 20µm. (*B*) *T. castaneum* brain from *Urn8* knockdown animal counterstained with DAPI (blue) showing complete elimination of DH37 (green) and DH47 (purple) expression in the *pars intercerebralis* (*PI*) in maximum projected z-stack. Scale bar = 50µm. *(C)* Relative transcript levels of *Urn8R*, *Urn8*, *Dh37* and *Dh47* gene expression relative to *Rp49* during control and gene knockdown conditions. (*D*) Wet weight (light bars) and dry weight (dark bars) of *Urn8R* depleted animals relative to control (Student's *t*-test; * $P < 0.05$; n=22-25).

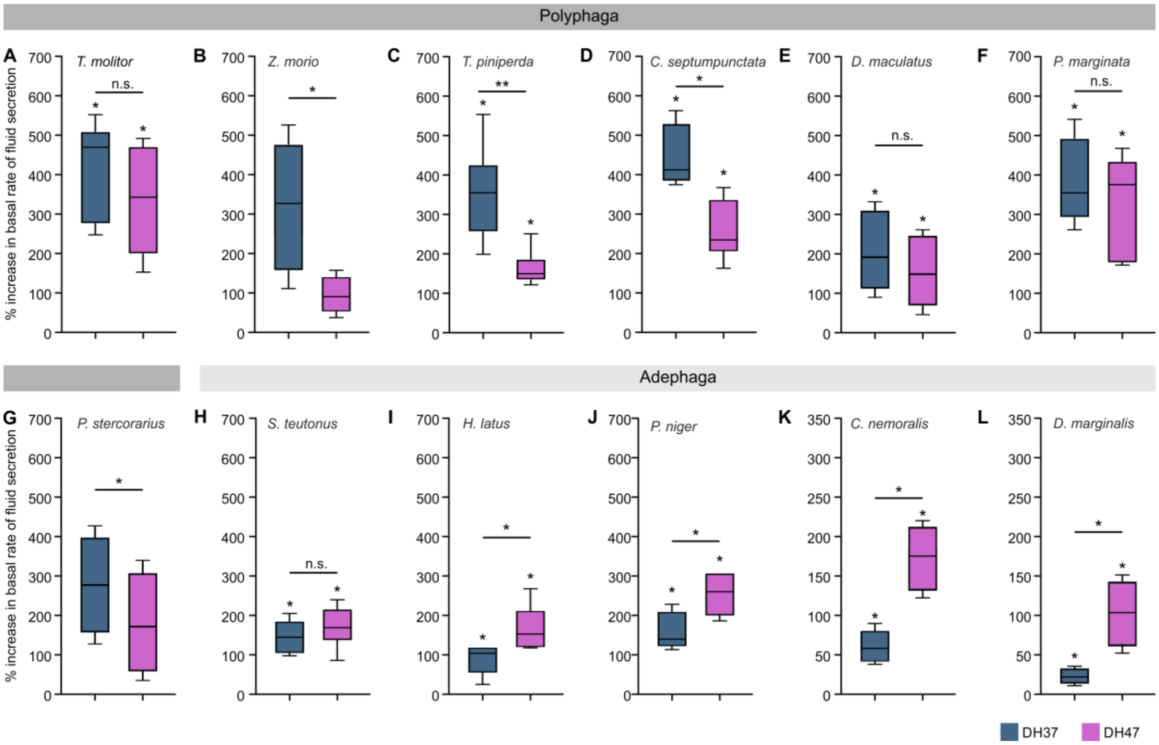

Fig S6. DH37-F and DH47-F binding predict diuretic activity across species. Box plots of percent increase in basal rates of fluid secretion following stimulation with DH37 and DH47 (10^{-7}M) on MTs from *(A) Tenebrio molitor* (n=10), *(B) Zophobas morio* (n=4-5) *(C) Tomicus piniperda* (n=7), *(D) Coccinella septumpunctata* (n=5), *(E) Dermestes maculatus* (n=4), *(F) Pachnoda marginata* (n=4-5), *(G) Platydracus stercorarius* (n=4), *(H) Stenolophus teutonus* (n=7), *(I) Harpalus latus* (n=6), *(J) Pterostichus niger* (n=7), *(K) Carabus nemoralis* (n=4), *(L) Dytiscus marginalis* (n=4)**.** Percent increase in fluid secretion rates following DH37 and DH47 stimulation compared to unstimulated controls (paired two-tailed Student's *t*-test; n.s. P > 0.05, * P < 0.05), and difference in DH37 and DH47 potency was tested (unpaired two-tailed Student's *t*-test n.s. P > 0.05, * P < 0.05, ** P < 0.01).

Table S1. Primer sequences used for In-Fusion cloning, RT-qPCR and dsRNA synthesis. Sequences marked in red correspond to vector sequence for In-Fusion cloning primers, and to the T7 promoter sequence for dsRNA primers.

**Cloning primer**	**Sequence**
Tc_Urn8RA_F	5'-CTCAAGCTTCGAATTATGAGTTGGTCCGAACCTCTCC-3'
Tc_Urn8RA_R	5'-GTCGACTGCAGAATTTTAACGGACGCTTCCGTTGAC-3'
Tc_Urn8RB_R	5'-GTCGACTGCAGAATTCTAAACGTTGCCTCCGCC-3'
**qPCR primer**	**Sequence**
Tc_Rp49_ F	5'-GTCTGACCGTTATGGCAAACTC-3'
Tc_Rp49_R	5'-TGTGCTTCGTTTTGGCATTGGAG-3'
Tc_Urn8_F	5'-GCCGCAAAATACAGACCTAACAGTG-3'
Tc_DH37_R	5'-TTTGGATACTTCGCTCGGAAGGCAC-3'
Tc_DH47_R	5'-CGGGATTCGGGGCTCAATTTATTC-3'
Tc_Urn8R_F	5'-TTATCACAATCTACGCCCCAACCCC-3'
Tc_Urn8R_R	5'-AATAGAGCGACAGTGAAGCCCTGTG-3'
**dsRNA primer**	**Sequence**
T7_Amp_R	5'-TAATACGACTCACTATAGGTTACCAATGCTTAATCAGTGAGGCACC-3'
T7_Amp_F	5'-TAATACGACTCACTATAGGATGAGTATTCAACATTTCCGTGTCGCCC-3'
T7_Dh47_R	5'-TAATACGACTCACTATAGGTTTTGGAGGCACTTGTGCCACTAC-3'
T7_Dh47_F	5'-TAATACGACTCACTATAGGTAAATTGAGCCCCGAATCCCGAAG-3'
T7_Dh37_R	5'-TAATACGACTCACTATAGGACTGTTTCTCACAGCGGTGTCTC-3'
T7_Dh37_F	5'-TAATACGACTCACTATAGGGTGCCTTCCGAGCGAAGTATC-3'
T7_Urn8_com_R	5'-TAATACGACTCACTATAGGCTGATTTTCGATGTCCTGCGATTCC-3'
T7_Urn8_com_F	5'-TAATACGACTCACTATAGGATGTGCCATCGTTTCGCCAAAACTG-3'

SI References

1. Halberg KA, Terhzaz S, Cabrero P, Davies SA, & Dow JA (2015) Tracing the evolutionary origins of insect renal function. *Nat Commun* 6:6800.
2. Trapnell C*, et al.* (2012) Differential gene and transcript expression analysis of RNA-seq experiments with TopHat and Cufflinks. *Nat Protoc* 7(3):562-578.
3. Richards S*, et al.* (2008) The genome of the model beetle and pest Tribolium castaneum. *Nature* 452(7190):949-955.
4. Li B*, et al.* (2008) Genomics, transcriptomics, and peptidomics of neuropeptides and protein hormones in the red flour beetle *Tribolium castaneum*. *Genome research* 18(1):113-122.
5. Egerod K*, et al.* (2003) Molecular cloning and functional expression of the first two specific insect myosuppressin receptors. *Proc Natl Acad Sci U S A* 100(17):9808-9813.
6. Maurer GW*, et al.* (2020) Analysis of genes within the schizophrenia-linked 22q11.2 deletion identifies interaction of night owl/LZTR1 and NF1 in GABAergic sleep control. *PLoS Genet* 16(4):e1008727.
7. Halberg KA*, et al.* (2016) The cell adhesion molecule Fasciclin2 regulates brush border length and organization in Drosophila renal tubules. *Nat Commun* 7.
8. Texada MJ*, et al.* (2019) A fat-tissue sensor couples growth to oxygen availability by remotely controlling insulin secretion. *Nat Commun* 10(1):1955.
9. Tabunoki H, Dittmer NT, Gorman MJ, & Kanost MR (2019) Development of a new method for collecting hemolymph and measuring phenoloxidase activity in *Tribolium castaneum*. *BMC research notes* 12(1):7.
10. Jourjine N, Mullaney BC, Mann K, & Scott K (2016) Coupled Sensing of Hunger and Thirst Signals Balances Sugar and Water Consumption. *Cell* 166(4):855-866.
11. Wiehart UI, Torfs P, Van Lommel A, Nicolson SW, & Schoofs L (2002) Immunocytochemical localization of a diuretic hormone of the beetle *Tenebrio molitor*, Tenmo-DH(37), in nervous system and midgut. *Cell and tissue research* 308(3):421-429.
12. Overend G*, et al.* (2015) A comprehensive transcriptomic view of renal function in the malaria vector, *Anopheles gambiae*. *Insect biochemistry and molecular biology* 67:47-58.
13. Cannell E*, et al.* (2016) The corticotropin-releasing factor-like diuretic hormone 44 (DH44) and kinin neuropeptides modulate desiccation and starvation tolerance in *Drosophila melanogaster*. *Peptides* 80:96-107.
14. O'Donnell MJ & Ruiz-Sanchez E (2015) The rectal complex and Malpighian tubules of the cabbage looper (*Trichoplusia* ni): regional variations in Na^+ and K^+ transport and cation reabsorption by secondary cells. *J Exp Biol* 218(Pt 20):3206-3214.
15. Kolosov D & O'Donnell MJ (2020) Mechanisms and regulation of chloride transport in the Malpighian tubules of the larval cabbage looper *Trichoplusia ni*. *Insect biochemistry and molecular biology* 116:103263.
